# Comprehensive Evaluation of Parameters Affecting One-Step Method for Quantitative Analysis of Fatty Acids in Meat

**DOI:** 10.3390/metabo9090189

**Published:** 2019-09-18

**Authors:** Michael P. Agnew, Cameron R. Craigie, Gayani Weralupitiya, Marlon M. Reis, Patricia L. Johnson, Mariza G. Reis

**Affiliations:** 1 AgResearch, Grassland Research Centre, Dairy Farm Road, Palmerston North 4472, New Zealand; marlon.dosreis@agresearch.co.nz; 2 AgResearch, Lincoln Research Centre, 1365 Spring Road, Lincoln 7674, New Zealand; cameron.craigie@agresearch.co.nz; 3 AgResearch, Ruakura Research Centre, 10 Bisley Road, Hamilton 3214, New Zealand; gayani_dw@yahoo.com; 4 Agresearch, Invermay Agricultural Centre, 176 Puddle Alley, Mosgiel 9092, New Zealand; tricia.johnson@agresearch.co.nz

**Keywords:** lamb, beef, venison, bimethylation, one-step transmethylation

## Abstract

Despite various direct transmethylation methods having been published and applied to analysis of meat fatty acid (FA) composition, there are still conflicting ideas about the best method for overcoming all the difficulties posed by analysis of complex mixtures of FA in meat. This study performed a systematic investigation of factors affecting a one-step method for quantitative analysis of fatty acids in freeze-dried animal tissue. Approximately 280 reactions, selected using factorial design, were performed to investigate the effect of temperature, reaction time, acid concentration, solvent volume, sample weight and sample moisture. The reaction yield for different types of fatty acids, including saturated, unsaturated (*cis*, *trans* and conjugated) and long-chain polyunsaturated fatty acids was determined. The optimised condition for one-step transmethylation was attained with four millilitres 5% sulfuric acid in methanol (as acid catalyst), four millilitres toluene (as co-solvent), 300 mg of freeze-dried meat and incubation at 70 °C for 2 h, with interim mixing by inversion at 30, 60 and 90 min for 15 s. The optimised condition was applied to meat samples from different species, covering a broad range of fat content and offers a simplified and reliable method for analysis of fatty acids from meat samples.

## 1. Introduction

Recently emphasis has been placed on animal-derived muscle (meat) fatty acid (FA) analysis, because correct estimation of FA composition is required to define not only nutrient composition of meat, but also to obtain an accurate determination of treatment effects that may alter FA composition [1,2,3,4,5].

Meat FA composition is affected by breed [6], and age [7] and can also be altered during animal production by making changes to the diets offered to the animals [8,9,10]. Overall, the target of such changes is to reduce the concentrations of saturated fatty acids (SFA) and increase those of polyunsaturated fatty acids (PUFA) [11]. These changes can alter the healthiness status of the meat by increasing the level of desirable PUFAs and can also alter other meat quality attributes such as flavour, tenderness and shelf life [11,12,13,14,15].

The most common process to analyse FAs is through the generation of fatty acids methyl esters (FAME) using an initial solvent extraction of lipids, followed by transesterification of the resulting lipids with an alcohol, typically methanol. In recent years, many researches have avoided the solvent extraction step by employing an in-situ transesterification process, in which FAs in a sample are simultaneously extracted and transesterified [4]. Some studies have indicated that this direct process leads to an increase in quantification precision compared with conventional solvent extraction–transesterification procedures [16]. However, because different types of samples may have different attributes, a large variation in reaction parameters are described in different studies, such as type of catalyst [17,18,19,20,21,22], use of co-solvent [19,22,23], polarity of the solvent [24], single- or double-phase reaction system [20,24], water content of the sample [17,21], reaction time [18,25] and temperature [16,23,26].

Therefore, the aim of this study was to investigate factors affecting the one-step method for quantitative analysis of FA in freeze-dried animal tissue. Previous studies have observed that nonpolar lipids such as triglycerides are not soluble in systems composed predominantly of methanol [24]. Therefore, in the present study, toluene was used as co-solvent, and the effect of temperature, reaction time, acid concentration, solvent volume, sample weight and sample moisture were evaluated. A systematic study was carried out to improve the understanding of the complex relationships between these different parameters and the performance of a one-step method for FA determination in meat samples.

## 2. Results and Discussion

**Figure 1:**
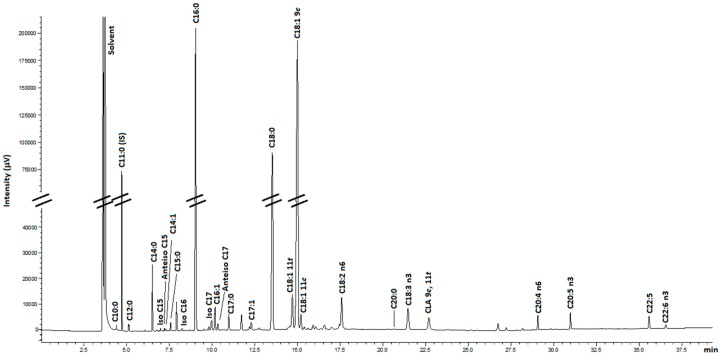
GC chromatogram representative meat FAMEs (fatty acids methyl esters). Legend. Methyl esters of: capric acid (C10:0), undecanoic acid (C11:0)—internal standard, lauric acid (12:0), myristic acid (C14:0), iso-pentadecanoic acid (Iso C15), anteiso-pentadecanoic acid (Anteiso C15), tetradecenoic acid (C14:1), pentadecanoic acid (C15:0), iso-hexadecanoic acid (Iso C16), palmitic acid (C16:0), iso-heptadecanoic acid (Iso C17), palmitoleic acid (C16:1), Margaric acid (C17:0), anteiso-heptadecanoic acid (Anteiso C17), heptadecenoic acid (C17:1), stearic acid (C18:0), *trans*-vaccenic acid (C18:1 11*t*), oleic acid (C18:1 9c) *cis*-vaccenic acid (C18:1 11*c*), linoleic acid (C18:2 n6), eicosanoic acid (C20:0), linolenic acid (C18:3 n3), *cis*-9, *trans*-11 conjugated linoleic acid (CLA 9*c*, 11*t*), arachidonic acid (C20:4), eicosapentaenoic acid (C20:5 n3), docosapentaenoic acid (C22:5), docosahexaenoic acid (C22:6 n3).

In the current study, different conditions of a one-step method for quantitative analysis of FAs in meat tissue were evaluated to investigate the effects of temperature, acid concentration, reaction time, as well as solvent volume, starting material weight and moisture content on direct transmethylation performance to generate FAMEs. For this purpose, the area of 27 FAMEs were monitored (supplementary material Table S1). In this research, the detailed effect of different conditions will be discussed using a representative group of FAs including medium- and long-chain saturated FAs [lauric acid (C12:0), palmitic acid (C16:0), and stearic acid (C18:0)], *cis* and *trans* unsaturated FAs [elaidic acid (C18:1 *t*9), *trans*- vaccenic acid (C18:1 *t*11) and oleic acid (C18:1 *c*9)], PUFAs [linoleic acid (C18:2 n6), linolenic acid (C18:3 n3), *cis*-9, *trans*-11 conjugated linoleic acid (CLA 9*c*, 11*t*)] and long-chain PUFAs [arachidonic acid (C20:4 n6), eicosapentaenoic acid (C20:5 n3), docosahexaenoic acid (C22:6 n3)]. A representative chromatogram of meat FAMEs separations with their respective peak identifications is showing in Figure 1.

### 2.1. Influence of Reaction Temperature

**Figure 2:**
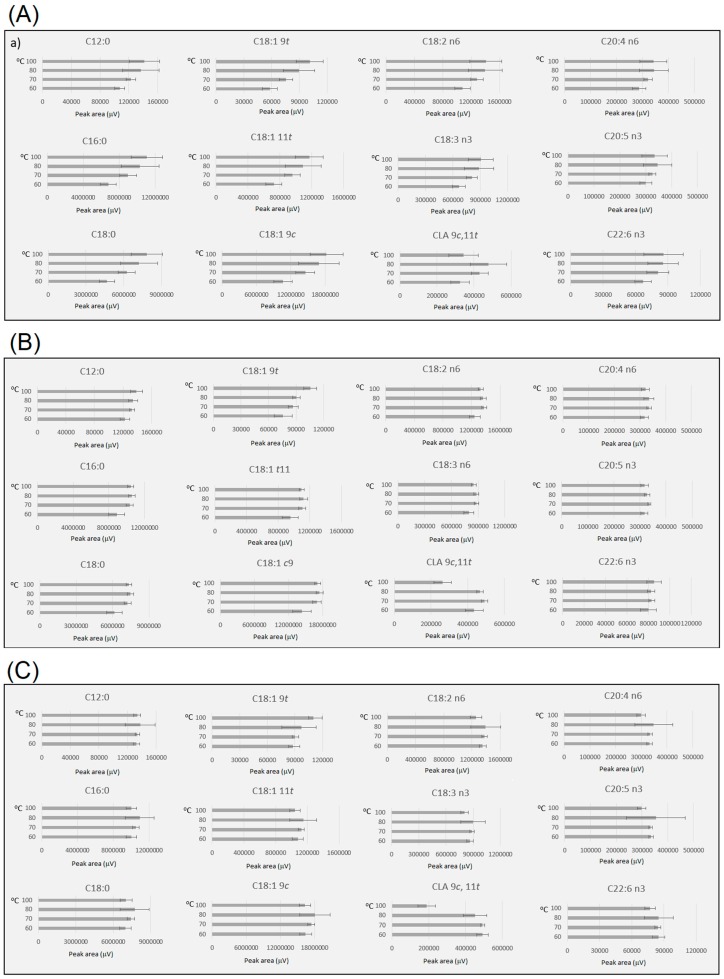
FAMEs yield (peak area normalised by volume of toluene and sample weight) comparison under different incubation temperatures and times (**a**)—1 h, (**b**)—2 h and (**c**)—3 h. Samples with acid concentration range from 3 to 7, total solvent volume of 8 and 10 mL, mass range from 200 to 400 mg and moisture between 0 and 7.5% were included to obtain the mean and standard deviation under the different conditions monitored. All experiments contained at least three replicates. For details, see Table S1.

Reaction temperature and time had a major effect on reaction efficiency (Figure 2). The results showed the intrinsic relationship between temperature and incubation time for acid-catalysed methanolysis, where changes in incubation conditions can lead to a profound impact in FAME yield and isomerisation rate. Incubation at 70 °C for 2 h was found to be an appropriated condition for one-step transmethylation procedure. The yield of several FAMEs, obtained by comparing peak areas of FAMEs, was lower when reaction temperature was 60 °C for 1 h or 2 h (Figure 2a,b). The lower efficiency in direct transmethylation at 60 °C was notable for long-chain FAs (C16:0, C18:0, C18:1 9c, C18:2 n6 and C18:3 n3). These results indicated that at 60 °C, lipids containing long-chain FAs were less susceptible to one-step methylation, which led to a lower yield, and this might have been caused by their lower solubility at 60 °C, triggering the reaction to require a much longer incubation time [27,28]. Indeed, the difference in yield of long-chain FAs at 60 °C disappeared at 3 h incubation (Figure 2c), while a similar yield of saturated FAMEs was observed at temperatures of 70 °C, 80 °C and 100 °C. Monounsaturated FAMEs also showed a similar yield under the different temperatures as observed for saturated FAMEs. However, an increase in the level of the *trans* FAME C18:1 9*t* was observed at 100 °C, suggesting that isomerisation of C18:1 9*c*, might take place at 100 °C. Additionally, the yield of PUFAs tended to decrease at 100 °C, and this decrease was prominent for CLA 9*c*, 11*t*, independent of the incubation time (1, 2 or 3 h).

It has been observed that direct acid-catalysed methylation of freeze-dried milk and CLA standards led to significant isomerization of CLA 9c, 11t at 70 °C using HCl-catalysed transmethylation [25]. However, previous studies observed that BF3-, HCl- and H2SO4 acid-catalysed transmethylation affect differently the isomerization/degradation of CLA isomers, with the biggest isomerisation caused by BF3, followed by HCl and H2SO4 [29]. While, as observed in the present study, incubation temperature and time also play an important role. Liu et al. [30], in a comparative study between the acid- and base-catalysed transesterification of dairy lipids, observed a decrease in the CLA 9c, 11t concentration corresponding to 11% and 35% loss using H2SO4 in methanol at 60 °C for 2 h and 6% H2SO4 in methanol at 80 °C for 1 h, respectively, compared with 0.2M KOH in methanol at 50 °C for 20 min. Similarly, a decrease of approximately 50% in the CLA c, t of dairy lipids was observed when using 5% HCl at 80 °C for 1 h by Kramer et al. [31]. However, milk differs as triglycerides correspond to the main proportion of lipid species [32] and are readily derivatised using base-catalysed derivatisation under mild conditions, whereas muscle tissue lipids contain significant amounts of phospholipids [5] including sphingolipids that are extremely resistant to alkaline treatment [33]. As a result, transmethylation of muscle lipids usually includes an acid treatment [8].

### 2.2. Influence of Acid Concentration

**Figure 3:**
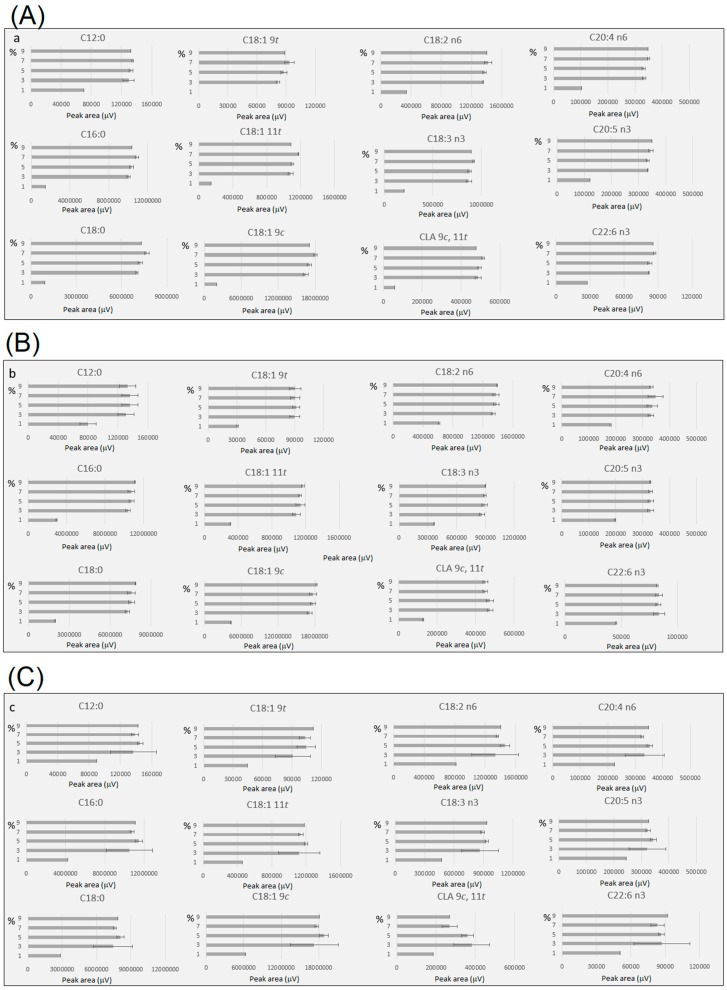
FAMEs yields (peak area normalised by volume of toluene and sample weight) comparison under different acid concentrations under different incubation conditions (**a**)—70 °C for 2 h, (**b**)—80 °C for 2 h and (**c**)—100 °C for 1 h. The samples with a total solvent volume of 8 and 10 mL, a mass range from 200 to 400 mg and moisture between 0 and 7.5% were included to obtain the mean and standard deviation under the different conditions monitored. All experiments contained at least three replicates, except for extreme conditions (acid concentrations of 1 and 9%), where one replicate was performed. For details see Table S1.

Figure 3 shows the yield of FAMEs using different H_2_SO_4_ concentrations (1 to 9%) under different incubation conditions (Figure 3a—70 °C for 2 h; Figure 3b—80 °C for 2 h; Figure 3c—100 °C for 1 h). The results indicated that once critical catalyst concentration was reached, reaction efficiency was not affected by increasing acid concentration. Reliable results were obtained with 5% sulfuric acid in methanol. Acid concentration of 1% and 2 h reaction time led to markedly lower yields of FAME, while acid concentration between 3% and 9% led to similar recoveries under different conditions of temperature and time.

### 2.3. Influence of Moisture Content

**Figure 4:**
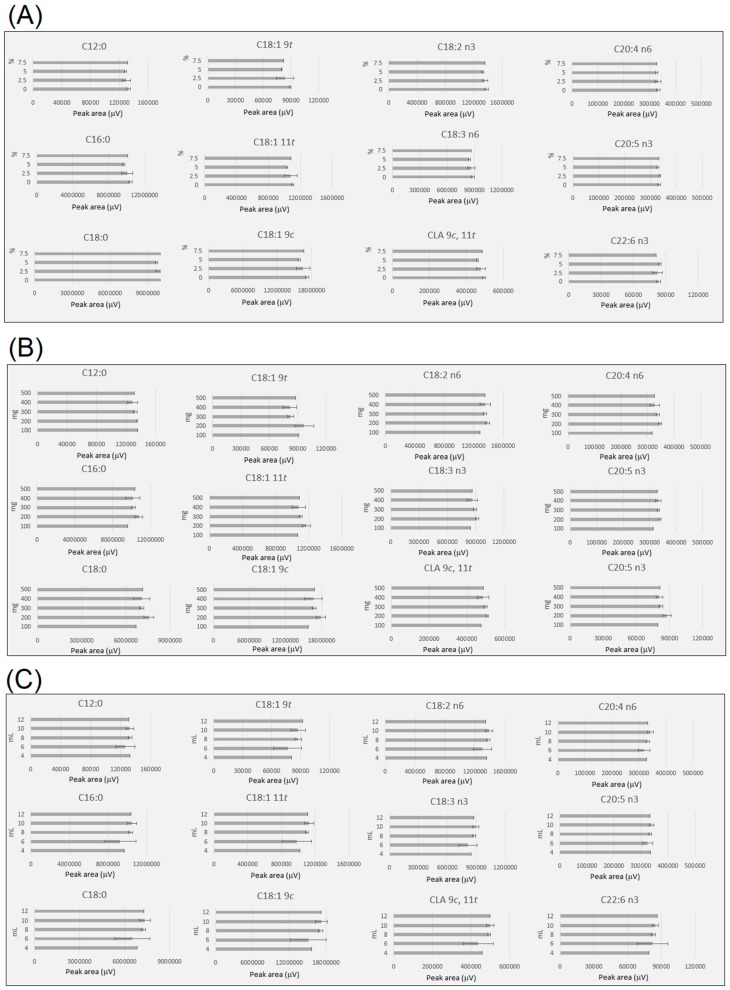
FAMEs yield (peak area normalised by volume of toluene and sample weight) comparison at 70 °C for 2 h under different conditions (**a**)—effect of moisture content; (**b**)—effect of sample weight and (**c**)—effect of total solvent volume, i.e., for 6 mL equal to 3 mL of H2SO4 in methanol and 3 mL of toluene). Samples with the acid concentration range from 3 to 7 were used to obtain the mean and standard deviation under the different conditions monitored. All experiments contained at least three replicates, except for extreme conditions (i.e., a—7.5% moisture; b—100 and 500 mg and c—4 mL of total solvent. For details see Table S1.

Previous studies have observed that water content can affect FAME formation [34], as acid-catalysed methanolysis is a reversible reaction, and free FA is formed during this process. To assess the effect of water content in the FAME yield under the conditions investigated in this study, water corresponding to 0, 2.5, 5 and 7.5% of the sample weight was added to the sample prior to incubation (Table S1). Figure 4a shows that moisture (0 to 7.5%) had an insignificant effect on the recovery of different fatty acids groups under three incubation conditions (60 °C for 3 h, 70 °C for 2 h and 80 °C for 2 h). Therefore, these results indicated that a low level of moisture (0 to 7.5%), corresponding to between 0 and 0.3% of water in the solvent mixture, does not hinder the FAMEs yield. These results are in agreement with previous studies that observed the nonsignificant effect of the addition of water, up to 2% of the reaction mixture, in the formation of FAME under acid-catalysed transmethylation [35].

### 2.4. Influence of Solvent Volume and Sample Weight

Solvent volume has also an important role in the efficiency of the FAME recovery, as lower reaction volumes can cause solvent saturation, while the amount of sample can also cause solvent saturation. Additionally, FA at low concentration ranges might not be detected using large solvent volumes or small sample sizes, due to sample dilution. Carrapiso et al. [24] observed that large amounts of sample underestimated the fat content in adipose tissues. In the case of the present study, which represented a lean meat, it was observed that different amounts of sample (200 mg to 500 mg) led to equivalent FAME yields (Figure 4b), however, a sample mass of 100 mg and a solvent volume of 8 mL led to a slightly lower yield of some FAMEs. Similarly, Carrapiso et al. [24] observed that too small sample size yields a high coefficient of variation. A solvent volume of 4 and 6 mL and sample weight of 200 to 400 mg led to slightly lower recovery at 70 °C for 2 h (Figure 4c). However, solvent volumes of 8, 10 and 12 mL were not significantly different for sample weights between 200 and 400 mg (Figure 4c). Therefore, a sample volume of 8 mL, corresponding to 4 mL of 5% sulfuric acid in methanol and 4 mL of toluene (as co-solvent), and sample size of 300 mg were selected for the optimised one-step method.

### 2.5. Statistical Significance of the Influence of the Different Parameters Investigated

Table 1 shows the statistical significance of the reaction conditions and their interaction on yield of different FAMEs monitored across the study. Acid concentration was the most important parameter that influenced the yield of different FAMEs during transmethylation, with 25 FAMEs yields affected by acid concentration, followed by temperature, with 24 FAMEs yields affected by temperature of incubation. While 21 FAMEs yields were affected by incubation time and solvent volume. Among the interactions of different parameters, the interaction between temperature and solvent volume was the most important parameter to influence FAME formation, followed by incubation time and solvent volume, and temperature and acid concentration.

### 2.6. Influence of the Salt Type

Sodium carbonate (NaCO_3_) has been used to neutralise acid-catalysed transmethylation [19], as the reaction of H_2_SO_4_ with sodium carbonate generates sodium sulphate, carbon dioxide and water. However, other studies have not included this neutralisation step [16,36]. These findings suggested that a neutralisation step might not be critical for acid-catalysed transesterification. In this study, it was observed that comparable FAME yields can be obtained by substituting sodium carbonate with sodium chloride (NaCl) (Table 2). The main advantage of using NaCl is the improvement in solvent separation (data not shown), due to the ability of saturated sodium chloride to inhibit emulsion formation.

### 2.7. Repeatability of the Optimised Direct Method for Quantitative Determination of Fatty Acid in Meat from Lamb, Beef and Venison

The repeatability of the direct method for muscle sample was evaluated using ten replicates of the same freeze-dried muscle from lamb, beef and venison (boneless lamb chumps with 2.8% of intramuscular fat (IMF), boneless lamb loin with 2.4% IMF, boneless beef with 3.34% IMF, boneless venison with 1.15% IMF and boneless lamb shoulder with 18.84% IMF). The results showed (Table 3) that direct method analysis of the different muscle types generated reproducible results, with RSDs below 10% for all main meat fatty acids monitored in the different samples (Table 3).

### 2.8. Comparative Evaluation Between the Optimised One-Step and Direct Bimethylation Procedures

**Figure 5:**
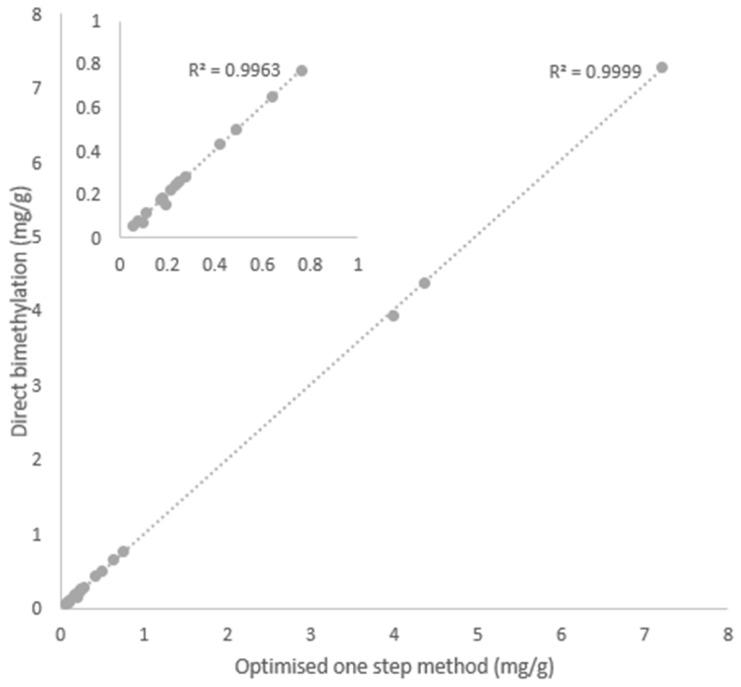
Correlation between one-step optimised method and the bimethylation procedure (mg/g of fresh muscle). Insert corresponds to FAs with concentration range between 0.1 to 1 mg/g of fresh muscle.

The one-step method using the optimized reaction conditions (for details see materials and methods) was compared with a direct bimethylation procedure, described by Lee et al. [37]. Figure 5 shows a high correlation (*R*^2^ = 0.99) between the analysis of a muscle sample using the bimethylation procedure and the optimised direct method (*n* = 10). The main advantages of the one-step method are the reduced number of steps and the ability to be performed under nonanhydrous conditions. These features are especially relevant in situations where many samples must be analysed.

## 3. Conclusions

The present study provided a comprehensive evaluation of the effect of different parameters on one-step method for direct analysis of FAs in meat. Among the different conditions evaluated, a simplified procedure was optimised. The optimised condition was achieved using 5% sulfuric acid in methanol (as acid catalyst), toluene (as co-solvent), freeze-dried meat and incubation at 70 °C for 2 h. The performance of the optimised method was comparable with the bimethylation method described previously, but has the advantage to have less steps and can be performed under nonanhydrous conditions. The direct method has many advantages including simplicity and speed. The method also showed to be applicable to meat samples from different species, covering a broad range of fat content. Altogether, the optimised one-step method increased the throughput of sample preparation for quantitative analysis of fatty acids in meat.

## 4. Materials and Methods

### 4.1. Reagents

Tridecanoin (tri C11:0) and FAME standards were purchased from Sigma-Aldrich, NZ. Sodium chloride (NaCl), HPLC-grade methanol, toluene and analytical-grade sulfuric acid (H_2_SO_4_) were sourced from Fisher (Themor Fisher Scientific, Auckland, NZ). Acetyl chloride and heptane were purchased from Sigma-Aldrich, NZ.

### 4.2. Samples

All meat samples used in this study were freeze-dried ground samples. A composite sample prepared by mixing lamb loin from different animals was used to investigate reaction conditions in the first part of this study, where 288 reactions were performed. After freeze-drying, samples were grounded to a fine powder using a commercial stainless-steel coffee grinder with 75 g hopper capacity (Brevielle, Auckland, NZ). Freeze-dried samples were stored at −20 °C, until analysis. The samples were weighed pre- and post-freeze-drying, and the moisture loss and total solids were calculated to convert back from dry weight basis to wet weight basis during normal analytical processes.

### 4.3. Experimental Design

This study aimed to identify a set of experimental conditions optimal for one-step method for quantitative analysis of fatty acids in meat. Six experimental factors were considered (Table 4): Temperature; reaction time; acid concentration; solvent volume; sample weight; and sample moisture. These are continuous factors, and they were assessed at four to five values. A full factorial, testing each factor at only three points would require 36 = 729 experiments. To reduce the number of experiments to a reasonable number, an approximating quadratic equation was fitted to a six-dimensional space with 28 parameters. The design points for fitting this quadratic equation are often chosen using a central composite design, which includes additional points to better estimate the parameters. Our previous experience suggested that temperature and reaction time would be the most important factors, and that the effects of these two variables would depend on each other. In order to obtain a good estimate of the combined inter-related effects of time and temperature, a full factorial of three times (1, 2, 3 h) by four temperatures (60 °C, 70 °C, 80 °C, 100 °C), giving a total of 12 combinations was chosen. The effects of the remaining four factors (acid concentration, solvent volume, sample weight and sample moisture) were thought to be less important and likely to act independently of each other, so the levels for these factors were chosen as a central composite design at each combination of temperature and time in the factorial design above. Each central composite design had 25 points. However, only four values of moisture (0%, 2.5%, 5%, 7.5%) were used, instead of the five values in a central composite design. The design was modified by omitting the central point and replacing the median values of the moisture by zero. This reduced the number of points in the central composite design to 24 and the total number of runs to 12 × 24 = 288. However, the results from eight experiments were removed because of problems with the analysis, consequently, the results of 280 experiments were used in this study.

### 4.4. FA Derivatisation using Bimethylation Procedure

Approximately 300 mg of the dried grounded samples were weighted out into a 15 mL kimax tube. Four millilitres 0.5 M sodium methoxide in anhydrous methanol and 1 mL heptane, containing 2 to 3 mg of Tri-C11 used as internal standard were added to each tube. The samples were tightly sealed with a Teflon-lined cap and vortexed well prior to heating for 15 min at 50 °C. Acetyl chloride in anhydrous methanol (1:10; *v*/*v*; 4 mL) was added before mixing thoroughly and heating for 1h at 60 °C. Heptane and distilled water were added, both at 2 mL, before mixing and centrifuging for 5 min at 1500× *g*. The organic solvent layer was pipetted into a second kimax tube before a further 2 mL of heptane was added to the original tube, mixed and centrifuged, as before. After pooling the organic layers in the second tube, anhydrous sodium sulphate (0.2 g) was added, mixed and centrifuged, as before, and an aliquot was used for GC analyses.

### 4.5. FA Derivatisation using Optimised One-Step Procedure

Samples were weighed into a kimax 15 mL tube (300 mg). Internal standard (tri C11) was added to the samples (3 mg of tri C11 in Toluene), followed by toluene (4 mL) and 5% sulfuric acid in methanol (4 mL). Samples were tightly sealed with a Teflon-lined cap and vortexed well before incubation at 70 °C for 2 h with interim mixing by inversion at 30, 60 and 90 min for 15 s. After cooling to room temperature for 20 min, saturated NaCl solution in distilled water was added to each tube (5 mL). Samples were shaken for 10 s, and then centrifuged at 1000× *g* for 10 min. An aliquot of the top layer was transferred into a 1.5 mL GC vial and analysed by GC-FID.

### 4.6. FAME Analysis

FAMEs, prepared as described above, were analysed with a GC-2010 (Shimadzu, Kyoto, Japan) and a RTX 2330 (90% biscyanopropyl—105m × 0.25 mm i.d × 0.2 µm film thickness) from Restek. The column temperature was kept at 175 °C for 17 min, then raised to 220 °C at a rate of 6 °C/min and held for 10 min. The carrier gas was hydrogen at a constant linear velocity of 50 cm/sec. The split ratio 50, injector temperature was 260 °C and detector 300 °C. An aliquot of 1 µL of the FAME sample, prepared as described before, was injected in the instrument. Identification of individual FAME isomers (including CLA and C18:1 isomers) was done by comparing with commercial standards.

### 4.7. Statistical Analysis

Second-order response surface regressions were fitted using Minitab 18 to identify effects of acid concentration (3, 5, 7, 9%), volume (2, 3, 4, 5, 6 mL), temperature (60, 70, 80, 100 °C) and time (1, 2, 3 h). *P* values lower than 0.05 were considered statistically significant (Table 1). Data were expressed as mean values with standard deviation (mean ± SD). Repeatability and comparative studies were repeated ten times (*n* = 10) (Table 2 and Table 3).

## Figures and Tables

**Table 1:** Summary of FA (fatty acid) yield responses using surface analyses of the different conditions evaluated.

Fatty Acids	*P*-values										*R*^2^-adj Values
-	Individual Effect				Interaction						
-	Temperature	Time	Acid Concentration	Volume	Temp * Time	Temp * Acid Concentration	Temp * Vol	Time * Acid Concentration	Time * Vol	Acid Concentration * Vol	
Total Fatty Acids	<0.001	<0.001	<0.001	0.005	<0.001	0.052	0.002	0.237	0.052	0.212	45.4%
C10:0	<0.001	<0.001	0.002	<0.001	<0.001	0.525	0.015	0.417	0.105	0.299	33.5%
C12:0	<0.001	<0.001	0.005	0.061	<0.001	0.679	0.086	0.488	0.184	0.546	26.5%
C14:0	<0.001	<0.001	<0.001	0.022	<0.001	0.067	<0.001	0.284	0.042	0.199	45.4%
Iso C15	<0.001	<0.001	0.004	<0.001	<0.001	0.071	0.239	0.503	0.348	0.400	42.6%
Anteiso C15	<0.001	<0.001	<0.001	<0.001	<0.001	0.066	0.036	0.346	0.031	0.558	43.5%
C15:0	<0.001	<0.001	<0.001	<0.001	<0.001	0.130	0.003	0.222	0.026	0.275	46.3%
Iso C16	<0.001	0.129	0.008	0.366	0.049	0.408	0.125	0.754	0.101	0.914	36.5%
C16:0	<0.001	<0.001	<0.001	0.005	<0.001	0.062	0.001	0.334	0.047	0.235	46.0%
Iso c17	0.041	<0.001	<0.001	<0.001	<0.001	0.019	<0.001	0.328	0.059	0.223	41.2%
C16:1	<0.001	<0.001	<0.001	0.002	<0.001	0.037	0.002	0.269	0.056	0.238	43.3%
Anteiso C17	<0.001	<0.001	0.001	0.010	<0.001	0.149	0.017	0.416	0.048	0.301	38.5%
C17:0	<0.001	<0.001	<0.001	<0.001	<0.001	0.063	0.001	0.310	0.050	0.219	45.9%
C17:1	<0.001	0.001	<0.001	0.001	<0.001	0.046	0.006	0.250	0.056	0.232	39.3%
C18:0	<0.001	<0.001	<0.001	0.011	<0.001	0.063	0.001	0.322	0.039	0.216	47.1%
C18:1 9*t*	<0.001	<0.001	<0.001	<0.001	<0.001	0.847	0.004	0.871	0.091	0.558	65.1%
C18:1 11*t*	<0.001	<0.001	<0.001	0.003	<0.001	0.093	<0.001	0.258	0.038	0.232	45.1%
C18:1 9*c*	<0.001	0.01	0.001	<0.001	<0.001	0.156	0.005	0.443	0.176	0.681	37.7%
C18:1 11*c*	<0.001	0.010	0.001	<0.001	<0.001	0.156	0.005	0.443	0.176	0.681	33.6%
C18:2 n6	<0.001	0.077	<0.001	0.111	<0.001	0.005	0.003	0.260	0.053	0.177	29.2%
C20:0	0.407	0.061	0.001	0.308	<0.001	0.254	0.055	0.541	0.735	0.636	4.7%
C18:3 n3	<0.001	0.014	<0.001	0.013	<0.001	0.013	0.002	0.269	0.041	0.198	33.5%
CLA 9*c*, 11*t*	<0.001	0.784	0.301	0.274	<0.001	<0.001	<0.001	0.282	0.066	0.333	38.3%
C20:4 n6	0.695	0.821	0.001	0.031	<0.001	0.003	0.039	0.123	0.271	0.084	17.5%
C20:5 n3	<0.001	0.554	0.856	0.934	0.906	0.819	0.443	0.660	0.861	0.729	24.7%
C22:5	0.543	0.739	<0.001	0.002	<0.001	0.001	0.006	0.385	0.011	0.112	23.0%
C22:6 n3	0.053	0.143	<0.001	0.067	<0.001	0.028	0.056	0.412	0.196	0.093	21.8%

**Table 2:** Comparative evaluation of the effect of Na_2_CO_3_ and NaCl on one-step optimised method for FA analysis (mg/g dry meat) (*n* = 10).

FAs	Na_2_CO_3_		NaCl	
	Mean ± STD	RSD	Mean ± STD	RSD
C10:0	0.21 ± 0.00	1.5	0.20 ± 0.00	2.4
C12:0	0.44 ± 0.01	1.6	0.44 ± 0.01	1.9
C14:0	3.81 ± 0.07	1.9	3.76 ± 0.07	1.9
iso C15	0.13 ± 0.00	2.8	0.13 ± 0.00	2.7
anteiso C15	0.19 ± 0.00	2.3	0.19 ± 0.01	3.6
C14:1	0.10 ± 0.00	1.7	0.10 ± 0.00	2.0
C15:0	0.45 ± 0.01	1.2	0.43 ± 0.01	2.2
iso C16	0.16 ± 0.01	3.8	0.16 ± 0.01	4.0
C16:0	34.26 ± 0.37	1.1	34.14 ± 0.38	1.1
iso C17	0.54 ± 0.01	1.5	0.53 ± 0.01	1.7
C16:1	1.60 ± 0.02	1.1	1.57 ± 0.02	1.5
anteiso C17	0.61 ± 0.01	2.2	0.59 ± 0.01	1.8
C17:0	1.44 ± 0.01	0.8	1.40 ± 0.02	1.5
C17:1	0.75 ± 0.00	0.6	0.73 ± 0.01	0.9
C18:0	24.17 ± 0.26	1.1	24.05 ± 0.27	1.1
C18:1 9*t*	0.31 ± 0.01	1.8	0.30 ± 0.01	3.1
C18:1 11*t*	3.77 ± 0.04	1.0	3.69 ± 0.06	1.8
C18:1 9*c*	56.32 ± 0.63	1.1	56.28 ± 0.47	0.8
C18:1 11*c*	1.39 ± 0.01	0.7	1.35 ± 0.01	1.0
C18:2 n6	4.70 ± 0.03	0.7	4.58 ± 0.04	0.9
C20:0	0.10 ± 0.00	1.5	0.10 ± 0.00	3.4
C18:3 n3	3.04 ± 0.02	0.5	2.98 ± 0.06	2.0
CLA 9c, 11*t*	1.72 ± 0.02	0.9	1.71 ± 0.08	4.6
C22	0.16 ± 0.00	1.4	0.15 ± 0.04	23.0
C20:4 n6	1.20 ± 0.02	1.7	1.17 ± 0.02	1.6
C22:1	0.10 ± 0.04	4.2	0.08 ± 0.01	15.9
C20:5 n3	1.17 ± 0.01	1.2	1.14 ± 0.01	1.2
C24:0	0.07 ± 0.01	11.5	0.07 ± 0.01	11.1
C24:1	0.06 ± 0.00	7.4	0.09 ± 0.02	27.3
C22:5	1.08 ± 0.01	1.3	1.06 ± 0.01	1.3
C22:6 n3	0.30 ± 0.01	2.5	0.29 ± 0.01	3.5
Unreported FA	10.48 ± 0.88	8.4	10.28 ± 0.85	8.3
Sum FA	154.85 ± 1.83	1.2	153.55 ± 1.87	1.2

STD standard deviation (*n* = 10); RSD Relative standard deviation (*n* = 10).

**Table 3:** FA composition (mg/g fresh meat) of meat cuts with different fat contents (boneless lamb chumps with 2.8% of intramuscular fat (IMF), boneless lamb loin with 2.4% IMF, boneless beef with 3.34% IMF, boneless venison with 1.15% IMF and boneless lamb shoulder with 18.84% IMF)), analysed using the optimised one-step direct transmethylation (*n* = 10).

FA	Lamb Chumps		Lamb Loin		Lamb Shoulder		Beef		Venison	
	Mean ± STD	RSD	Mean ± STD	RSD	Mean ± STD	RSD	Mean ± STD	RSD	Mean ± STD	RSD
C14:0	0.84 ± 0.01	1.25	0.70 ± 0.01	1.47	8.36 ± 0.27	3.18	0.64 ± 0.01	1.34	0.21 ± 0.01	4.38
C14:1	0.03 ± 0.00	4.08	0.02 ± 0.00	5.09	0.22 ± 0.02	8.77	0.18 ± 0.00	2.53	0.06 ± 0.00	3.74
C15:0	0.13 ± 0.00	1.59	0.10 ± 0.00	2.72	1.29 ± 0.04	3.39	0.13 ± 0.00	2.31	0.05 ± 0.00	5.06
C16:0	5.61 ± 0.06	1.14	5.12 ± 0.04	0.69	42.21 ± 1.22	2.89	7.50 ± 0.11	1.46	1.67 ± 0.04	2.69
C16:1	0.28 ± 0.01	2.40	0.24 ± 0.01	2.87	2.01 ± 0.06	3.03	0.90 ± 0.01	1.17	0.32 ± 0.01	3.16
C17:0	0.30 ± 0.01	1.77	0.25 ± 0.00	1.41	2.91 ± 0.09	3.07	0.31 ± 0.01	1.86	0.06 ± 0.00	5.52
C17:1	0.14 ± 0.00	1.96	0.12 ± 0.00	2.28	1.01 ± 0.03	3.10	0.25 ± 0.00	1.62	0.04 ± 0.00	7.28
C18:0	4.44 ± 0.05	1.12	3.88 ± 0.02	0.63	35.39 ± 0.92	2.61	4.47 ± 0.06	1.33	1.54 ± 0.03	1.86
C18:1 11*t*	0.80 ± 0.02	1.89	0.69 ± 0.02	3.23	7.86 ± 0.30	3.79	0.37 ± 0.01	2.09	0.12±0.01	5.93
C18:1 9*c*	7.68 ± 0.09	1.21	7.34 ± 0.06	0.81	53.06 ± 1.37	2.58	12.07 ± 0.15	1.26	1.61 ± 0.04	2.46
C18:1 11*c*	0.34 ± 0.00	1.34	0.29 ± 0.01	4.68	1.90 ± 0.09	4.61	0.55 ± 0.01	2.71	0.37 ± 0.01	1.85
C18:2 n6	1.59 ± 0.02	1.00	1.12 ± 0.02	1.52	4.57 ± 0.18	3.96	0.68 ± 0.01	1.07	1.30 ± 0.02	1.51
C18:3 n3	0.88 ± 0.01	1.05	0.64 ± 0.01	1.04	3.67 ± 0.11	2.95	0.42 ± 0.01	2.19	0.51 ± 0.01	2.19
CLA 9*c*, 11*t*	0.29 ± 0.01	2.09	0.28 ± 0.01	2.66	2.40 ± 0.11	4.39	0.19 ± 0.01	4.20	0.04 ± 0.00	9.07
C20:4 n6	0.34 ± 0.00	1.00	0.27 ± 0.00	1.12	0.37 ± 0.02	4.91	0.25 ± 0.00	1.56	0.49 ± 0.00	0.98
C20:5 n3	0.24 ± 0.00	0.76	0.22 ± 0.00	1.33	0.23 ± 0.02	7.01	0.18 ± 0.00	1.81	0.35 ± 0.01	1.62
C22:5	0.22 ± 0.00	1.73	0.20 ± 0.00	2.15	0.39 ± 0.02	5.44	0.23 ± 0.00	1.92	0.32 ± 0.01	1.59
C22:6 n3	0.07 ± 0.00	2.60	0.07 ± 0.00	4.38	<0.15	-	0.04 ± 0.00	9.95	0.09 ± 0.00	5.06
Unreported FA	2.77 ± 0.12	4.34	2.26 ± 0.08	3.35	14.44 ± 0.55	3.81	2.52 ± 0.07	2.85	1.86 ± 0.05	2.45
SUM FA	26.97 ± 0.37	1.36	23.81 ± 0.20	0.82	182.37 ± 4.95	2.71	31.86 ± 0.39	1.21	11.03 ± 0.20	1.77

STD standard deviation (*n* = 10); RSD Relative standard deviation (*n* = 10).

**Table 4:** Factors and levels used for the experimental design.

Variables	Levels Used					Optimum Conditions
Temperature (°C)	60	70	80	100		70
Time (hr)	1	2	3			2
Concentration (% H_2_SO_4_ in methanol)	3	5	7	9		5
Acid and toluene volume (mL)	2	3	4	5	6	4 (each)
Sample weight (mg)	100	200	300	400	500	300
Moisture (%)	0	2.5	5	7.5		0–7.5%

## Supplementary Materials

The following are available online at https://www.mdpi.com/2218-1989/9/9/189/s1, Table S1: Fatty acids peak area normalised by the volume (toluene) and sample weight (TP = temperature; TM = time; CO = acid concentration; VT = volume toluene; VA = volume acid; M = sample weight; H =moisture; C11 = area of the internal standard not normalised; C11N = area of the internal standard normalised by the volume of toluene).

## References

[1] Jenkins T.C. (2010). Technical note: Common analytical errors yielding inaccurate results during analysis of fatty acids in feed and digesta samples. J. Dairy Sci..

[2] Pena R.N., Noguera J.L., García-Santana M.J., González E., Tejeda J.F., Ros-Freixedes R., Ibáñez-Escriche N. (2019). Five genomic regions have a major impact on fat composition in Iberian pigs. Sci. Rep..

[3] Dugan M.E.R., Wood J. (2018). Letter to the editor. Meat Sci..

[4] De Paola E.L., Montevecchi G., Masino F., Antonelli A., Fiego D.P.L. (2017). Single step extraction and derivatization of intramuscular lipids for fatty acid Ultra Fast GC analysis: Application on pig thigh. J. Food Sci. Technol..

[5] Wood J., Enser M., Fisher A., Nute G., Sheard P., Richardson R., Hughes S., Whittington F. (2008). Fat deposition, fatty acid composition and meat quality: A review. Meat Sci..

[6] Bhuiyan M.S.A., Lee D.H., Kim H.J., Lee S.H., Cho S.H., Yang B.S., Kim S.D. (2018). Estimates of genetic parameters for fatty acid compositions in the longissimus dorsi muscle of Hanwoo cattle. Animal.

[7] Belaunzaran X., Lavín P., Mantecón A.R., Kramer J.K.G., Aldai N. (2018). Effect of slaughter age and feeding system on the neutral and polar lipid composition of horse meat. Animal.

[8] Vahmani P., Rolland D., McAllister T., Block H., Proctor S., Guan L., Prieto N., López-Campos Ó., Aalhus J., Dugan M. (2017). Effects of feeding steers extruded flaxseed on its own before hay or mixed with hay on animal performance, carcass quality, and meat and hamburger fatty acid composition. Meat Sci..

[9] Schiavon S., Bergamaschi M., Pellattiero E., Simonetto A., Tagliapietra F. (2017). Fatty Acid Composition of Lamb Liver, Muscle, And Adipose Tissues in Response to Rumen-Protected Conjugated Linoleic Acid (CLA) Supplementation Is Tissue Dependent. J. Agric. Food Chem..

[10] Jaturasitha S., Chaiwang N., Kayan A., Kreuzer M. (2016). Nutritional strategies to improve the lipid composition of meat, with emphasis on Thailand and Asia. Meat Sci..

[11] Wood J., Richardson R., Nute G., Fisher A., Campo M.M., Kasapidou E., Sheard P., Enser M. (2004). Effects of fatty acids on meat quality: A review. Meat Sci..

[12] Raes K. (2003). Meat quality, fatty acid composition and flavour analysis in Belgian retail beef. Meat Sci..

[13] Arshad M.S., Sohaib M., Ahmad R.S., Nadeem M.T., Imran A., Arshad M.U., Kwon J.-H., Amjad Z. (2018). Ruminant meat flavor influenced by different factors with special reference to fatty acids. Lipids Heal. Dis..

[14] Flakemore A.R., Malau-Aduli B.S., Nichols P.D., Malau-Aduli A.E.O. (2017). Omega-3 fatty acids, nutrient retention values, and sensory meat eating quality in cooked and raw Australian lamb. Meat Sci..

[15] Scollan N., Hocquette J.-F., Nuernberg K., Dannenberger D., Richardson I., Moloney A. (2006). Innovations in beef production systems that enhance the nutritional and health value of beef lipids and their relationship with meat quality. Meat Sci..

[16] Abdulkadir S., Tsuchiya M. (2008). One-step method for quantitative and qualitative analysis of fatty acids in marine animal samples. J. Exp. Mar. Boil. Ecol..

[17] O’Fallon J.V., Busboom J.R., Nelson M.L., Gaskins C.T. (2007). A direct method for fatty acid methyl ester synthesis: Application to wet meat tissues, oils, and feedstuffs. J. Anim. Sci..

[18] Murrieta C., Hess B., Rule D. (2003). Comparison of acidic and alkaline catalysts for preparation of fatty acid methyl esters from ovine muscle with emphasis on conjugated linoleic acid. Meat Sci..

[19] Sukhija P.S., Palmquist D.L. (1988). Rapid method for determination of total fatty acid content and composition of feedstuffs and feces. J. Agric. Food Chem..

[20] Araujo P., Nguyen T.-T., Frøyland L., Wang J., Kang J.X. (2008). Evaluation of a Rapid Method for the Quantitative Analysis of Fatty Acids in Various Matrices. J. Chromatogr. A.

[21] Griffiths M.J., Van Hille R.P., Harrison S.T.L., Griffiths M., Hille R.P. (2010). Selection of Direct Transesterification as the Preferred Method for Assay of Fatty Acid Content of Microalgae. Lipids.

[22] Castro-Gómez P., Fontecha J., Rodríguez-Alcalá L.M. (2014). A high-performance direct transmethylation method for total fatty acids assessment in biological and foodstuff samples. Talanta.

[23] Hidalgo P., Ciudad G., Schober S., Mittelbach M., Navia R. (2015). Improving the FAME Yield of in Situ Transesterification from Microalgal Biomass through Particle Size Reduction and Cosolvent Incorporation. Energy Fuels.

[24] Carrapiso A.I., Timón M.L., Petrón M.J., Tejeda J.F., García C. (2000). In situ transesterification of fatty acids from Iberian pig subcutaneous adipose tissue. Meat Sci..

[25] Lee M.R.F., Tweed J.K.S. (2008). Isomerisation of cis-9 trans-11 conjugated linoleic acid (CLA) to trans-9 trans-11 CLA during acidic methylation can be avoided by a rapid base catalysed methylation of milk fat. J. Dairy Res..

[26] Juarez M., Polvillo O., Contò M., Ficco A., Ballico S., Failla S. (2008). Comparison of four extraction/methylation analytical methods to measure fatty acid composition by gas chromatography in meat. J. Chromatogr. A.

[27] Hoerr C.W., Harwood H.J. (1951). Solubilities of high molecular weight aliphatic compounds in n-hexane. J. Org. Chem..

[28] Crompton M.J., Dunstan R.H. (2018). Evaluation of in-situ fatty acid extraction protocols for the analysis of staphylococcal cell membrane associated fatty acids by gas chromatography. J. Chromatogr. B.

[29] Yamasaki M., Kishihara K., Ikeda I., Sugano M., Yamada K. (1999). A recommended esterification method for gas chromatographic measurement of conjugated linoleic acid. J. Am. Oil Chem. Soc..

[30] Liu Z., Ezernieks V., Rochfort S., Cocks B. (2018). Comparison of methylation methods for fatty acid analysis of milk fat. Food Chem..

[31] Kramer J.K.G., Fellner V., Dugan M.E.R., Sauer F.D., Mossoba M.M., Yurawecz M.P. (1997). Evaluating acid and base catalysts in the methylation of milk and rumen fatty acids with special emphasis on conjugated dienes and total trans fatty acids. Lipids.

[32] Liu Z., Moate P., Rochfort S. (2019). A simplified protocol for fatty acid profiling of milk fat without lipid extraction. Int. Dairy J..

[33] MacGee J., Williams M.G. (1981). Prepartion of sphingolipid fatty acid methyl esters for determination by gas—liquid chromatography. J. Chromatogr. A.

[34] Dong T., Yu L., Gao D., Yu X., Miao C., Zheng Y., Lian J., Li T., Chen S. (2015). Direct quantification of fatty acids in wet microalgal and yeast biomass via a rapid in situ fatty acid methyl ester derivatization approach. Appl. Microbiol. Biotechnol..

[35] Ulberth F., Henninger M. (1992). One-step extraction/methylation method for determining the fatty acid composition of processed foods. J. Am. Oil Chem. Soc..

[36] Ken’ichi I., Yumeto F. (2010). Preparation of fatty acid methyl esters for gas-liquid chromatography. J. Lipid Res..

[37] Lee M., Tweed J., Kim E., Scollan N. (2012). Beef, chicken and lamb fatty acid analysis—A simplified direct bimethylation procedure using freeze-dried material. Meat Sci..

