# Supplementary material for: Comprehensive Evaluation of Parameters Affecting One-Step Method for Quantitative Analysis of Fatty Acids in Meat
Source: Metabolites. 2019 Sep 18;9(9):189. doi: 10.3390/metabo9090189 (PMC6780067; doi:10.3390/metabo9090189)
Supplement: Supplementary file 1 [file metabolites-09-00189-s001.pdf]

Supplementary material

Table S1. Fatty acids peak area normalised by the volume (toluene) and sample weight (TP = temperature; TM=time; CO= acid concentration; VT= volume toluene; VA = volume acid; M= sample weight; H=moisture; C11= area of the internal standard not normalised; C11N=area of the internal standard normalised by the volume of toluene).

#	TP	TM	CO	VT	VA	M	H	C11:0	C11 N	total	unreported	C10:0	C12:0	C14:0	iso C15	anteiso C15
1	60	1	5	4	4	100	0	205042	820168	34354049	1633289	53040	125760	851520	0	0
2	60	3	5	4	4	100	0	246406	985624	42311303	2085402	65426	138812	1056000	0	55446
3	60	1	3	3	3	200	2.5	163332	489996	24036141	1752574	38134	106701	570209	0	29806
4	60	1	7	5	5	200	2.5	163473	817365	37035381	2181386	52770	120074	895711	0	49044
5	60	2	3	5	5	200	2.5	171032	855160	37686606	2086581	53596	121355	913695	0	46281
6	60	3	3	3	3	200	2.5	280612	841836	38254635	2223113	56093	124624	929005	39761	57322
7	60	3	7	5	5	200	2.5	207527	1037635	47178606	2552881	63875	138825	1149625	38125	60850
8	60	1	3	5	5	200	5	116827	584135	27227194	1681585	43268	103293	641268	0	42439
9	60	1	7	3	3	200	5	246202	738606	30809549	2005905	46302	116605	723512	28244	41341
10	60	2	7	5	5	200	5	196960	984800	42314747	2237011	60796	125448	1031741	33383	53756
11	60	3	3	5	5	200	5	187562	937810	42193902	2398975	62230	132819	1040417	39485	48578
12	60	3	7	3	3	200	5	335300	1005900	43874344	2588329	59839	133990	1074234	37595	52946
13	60	1	5	4	4	300	7.5	148450	593800	28000532	1963515	39652	102849	640548	20254	35037
14	60	2	5	4	4	300	7.5	213321	853284	38775869	2355354	53677	121017	933835	29690	46693
15	60	3	5	4	4	300	7.5	234867	939468	44397578	2749551	58592	130039	1082882	38237	51250

16	60	1	5	4	4	300	0	172946	691784	32448902	2109154	45701	114684	773223	26963	40811
17	60	1	5	4	4	300	0	164767	659068	30800207	2045820	44627	106813	716907	26133	37747
18	60	1	5	4	4	300	0	163976	655904	31024203	2178716	45684	109368	729908	20750	35842
19	60	1	5	4	4	300	0	171397	685588	30734898	2075139	45980	107666	714381	30247	40134
20	60	1	1	4	4	300	0	94218	376872	19702021	1610734	31248	91248	425769	0	24977
21	60	1	9	4	4	300	0	136382	545528	23209137	1864211	31396	95933	487839	0	29651
22	60	1	5	2	2	300	0	197270	394540	19980213	1679408	29685	91275	424497	13685	25523
23	60	1	5	6	6	300	0	118726	712356	33502429	2099577	47644	111980	798614	28040	41129
24	60	2	5	4	4	300	0	223717	894868	41629387	2555199	58215	127020	1019651	34228	52537
25	60	2	5	4	4	300	0	226937	907748	42509902	2469598	57848	128119	1042416	36092	56937
26	60	2	5	4	4	300	0	227292	909168	41179763	2545861	57928	126767	1000341	37836	55948
27	60	2	1	4	4	300	0	165276	661104	31250030	2033363	44797	112366	736680	31948	47412
28	60	2	9	4	4	300	0	193885	775540	31699653	2158984	44603	110479	733705	23489	39436
29	60	2	5	6	6	300	0	156695	940170	43630828	2557177	58401	128428	1064467	33474	52164
30	60	3	5	4	4	300	0	242484	969936	46106614	2844830	62780	134177	1149521	46426	61810
31	60	3	5	4	4	300	0	242708	970832	44586759	2624519	59133	131560	1099093	37507	53573
32	60	3	5	4	4	300	0	240375	961500	44442211	2662775	60148	133383	1085463	40738	63262
33	60	3	5	4	4	300	0	238536	954144	45257638	2581920	60027	135007	1109960	43799	64913
34	60	3	1	4	4	300	0	198877	795508	36686441	2252282	51497	117523	884596	30728	43894

35	60	3	9	4	4	300	0	230923	923692	39934391	2553639	53477	121597	949785	31745	47329
36	60	3	5	2	2	300	0	424889	849778	38075867	2510900	52262	123503	920268	31497	47832
37	60	3	5	6	6	300	0	162472	974832	48737933	2811762	65250	138868	1225480	47191	56526
38	60	1	3	5	5	400	2.5	86344	431720	23019942	1771894	34874	95768	508577	16940	28186
39	60	1	7	3	3	400	2.5	170745	512235	24053003	1861847	34418	94515	523775	19790	31983
40	60	2	3	3	3	400	2.5	146661	439983	22881242	1820820	31948	95919	501563	16052	27400
41	60	3	3	5	5	400	2.5	161403	807015	38727620	2325039	54140	121920	932843	37307	53641
42	60	3	7	3	3	400	2.5	310670	932010	42612475	2710385	55925	126744	1041947	34383	52128
43	60	1	3	3	3	400	5	75574	226722	13989500	1584931	20939	80689	276076	0	17470
44	60	1	7	5	5	400	5	140579	702895	32283733	2218004	44435	107362	735427	23128	36972
45	60	2	3	5	5	400	5	133158	665790	31584791	2145662	44659	108447	739179	22803	34646
46	60	2	7	3	3	400	5	250507	751521	33159434	2302055	45935	112481	786815	25347	38147
47	60	3	3	3	3	400	5	188915	566745	26969737	1920589	37963	102044	612689	21163	32348
48	60	3	7	5	5	400	5	195420	977100	46984062	2882793	61206	131495	1132450	42651	61520
49	60	1	5	4	4	500	0	139081	556324	27234721	2070088	39431	101844	606966	22060	35263
50	60	2	5	4	4	500	0	199675	798700	38193506	2566927	52129	118699	909920	29952	47398
51	60	3	5	4	4	500	0	204230	816920	48027490	3153702	64032	145916	1161996	39479	56850
52	70	1	5	4	4	100	0	235758	943032	42233988	3900474	59223	130408	1003534	0	53786
53	70	1	3	5	5	200	2.5	171736	858680	39207743	3389893	54825	121200	917625	31300	48200

54	70	1	7	5	5	200	5	192126	960630	44274838	3499105	58333	130606	1049242	37222	60556
55	70	2	5	2	2	300	0	460904	921808	43064391	2824913	58574	132106	1059789	33802	51941
56	70	1	5	4	4	300	7.5	200528	802112	36854535	3009407	49799	118040	864148	29248	44564
57	70	1	7	3	3	200	2.5	308172	924516	42733596	3758637	57905	128472	1011648	33663	50819
58	70	2	3	3	3	200	2.5	314345	943035	43059102	3367441	57779	130279	1028103	34235	56353
59	70	2	7	5	5	200	2.5	202855	1014275	48868417	3944067	63125	135650	1168250	41925	59100
60	70	3	7	3	3	200	2.5	340176	1020528	46420722	2851139	62045	136537	1137657	38970	55925
61	70	1	3	3	3	200	5	232409	697227	31286541	2511762	44864	112492	720362	21844	38698
62	70	1	5	4	4	300	0	218340	873360	41026487	3138300	55518	123839	982836	33017	51799
63	70	2	3	5	5	200	5	197146	985730	45497256	3130191	61244	134652	1101716	36244	55473
64	70	2	7	3	3	200	5	337236	1011708	47549038	3444219	63348	137045	1150758	40652	60879
65	70	3	3	3	3	200	5	324839	974517	44249628	2991787	61797	133634	1092223	36416	53183
66	70	3	5	2	2	300	0	479396	958792	44838865	3069113	58148	130517	1099383	35631	53725
67	70	3	3	5	5	200	2.5	197379	986895	43739115	2361943	61515	131187	1075051	36894	54394
68	70	3	7	5	5	200	5	200051	1000255	47373997	2598922	63675	138550	1163925	40675	60500
69	70	1	5	4	4	300	0	220765	883060	40590686	2554419	55120	124293	980720	35000	52827
70	70	2	5	4	4	100	0	245930	983720	44348258	4266493	65294	136275	1049725	0	56784
71	70	3	5	4	4	100	0	241327	965308	45162392	4366969	65769	137462	1075654	0	56115
72	70	1	5	2	2	300	0	321387	642774	29859318	2715299	41479	107076	668119	22535	37551

73	70	1	5	6	6	300	0	154747	928482	44448956	3487055	60947	128645	1069086	31026	53368
74	70	1	5	4	4	300	0	215476	861904	41666050	3265945	55331	124918	1001141	32472	49744
75	70	1	5	4	4	300	0	227420	909680	43083255	3772914	56763	128535	1019010	34368	50528
76	70	1	7	5	5	400	2.5	188037	940185	45747663	3252038	58425	127663	1095025	38013	58275
77	70	1	3	5	5	400	5	130748	653740	32302218	2880283	44206	108486	731638	24256	38524
78	70	1	1	4	4	300	0	12845	51380	6093834	1701635	0	63734	97980	0	0
79	70	1	9	4	4	300	0	224658	898632	40378975	3219895	53640	123547	948453	33227	48907
80	70	2	5	4	4	300	7.5	240603	962412	45451646	3284878	59921	131593	1104407	36761	57731
81	70	3	5	4	4	300	7.5	252181	1008724	47256293	3372491	61545	134310	1133993	39446	59894
82	70	2	5	4	4	300	0	242760	971040	45755382	3309907	60106	129595	1100784	37090	57887
83	70	2	5	4	4	300	0	245360	981440	47663477	3838344	61293	134547	1137387	37213	54547
84	70	2	5	4	4	300	0	246004	984016	46119260	3028863	61245	132331	1118013	39656	60358
85	70	2	5	4	4	300	0	250738	1002952	48267744	4221426	62980	135894	1153669	38291	59616
86	70	2	1	4	4	300	0	19803	79212	7889881	1907161	0	69954	125769	0	0
87	70	2	9	4	4	300	0	246389	985556	46079082	2887360	59894	132278	1110901	38477	60013
88	70	3	5	4	4	300	0	245786	983144	46769803	3502055	60848	132026	1128993	39113	57377
89	70	3	5	4	4	300	0	249414	997656	47857781	3380351	61836	136302	1163226	40262	59921
90	70	1	3	3	3	400	2.5	137642	412926	22234497	2411299	31708	93364	466923	14911	25745
91	70	1	5	4	4	500	0	178108	712432	35431624	2452923	48152	112697	845651	27527	45788

92	70	2	3	5	5	400	2.5	176052	880260	44266943	3055448	57198	124309	1064309	34661	54975
93	70	2	7	3	3	400	2.5	318495	955485	44855888	3012846	57756	129292	1093788	35843	54464
94	70	3	3	3	3	400	2.5	267350	802050	38097136	2508852	51246	118761	920052	30522	46500
95	70	3	7	5	5	400	2.5	203265	1016325	50219429	3699318	62184	134888	1201625	41625	63226
96	70	1	7	3	3	400	5	261173	783519	35463327	2733702	47716	114905	827454	26768	41491
97	70	2	3	3	3	400	5	215714	647142	31362814	2566981	43332	108075	718234	24112	36030
98	70	2	7	5	5	400	5	200130	1000650	49002788	3008522	62152	134055	1185659	38595	57264
99	70	3	3	5	5	400	5	190054	950270	46045472	3828484	59125	129075	1098775	38325	55688
100	70	3	7	3	3	400	5	328968	986904	47544445	3573214	59642	133269	1159030	37075	56343
101	70	3	5	4	4	300	0	245726	982904	47638644	4096824	61103	132678	1135322	38937	58658
102	70	3	5	4	4	300	0	248065	992260	48768653	3546799	62967	136689	1181629	38689	57589
103	70	3	9	4	4	300	0	275002	1100008	53332842	3918882	66320	152480	1275933	45160	67280
104	70	2	5	4	4	500	0	240652	962608	46567691	3274875	59488	131664	1133056	36152	55328
105	70	3	1	4	4	300	0	32874	131496	10561084	1951058	0	73638	182188	0	0
106	70	3	5	4	4	500	0	245509	982036	47563356	3341745	59727	132345	1157868	37555	56104
107	70	2	5	6	6	300	0	161009	966054	47517559	4286988	62144	131016	1114289	40033	55298
108	70	3	5	6	6	300	0	163812	982872	48279858	2747687	64839	135482	1196459	38164	58898
109	80	2	5	4	4	100	0	256967	1027868	47630348	5061828	65840	144200	1126240	0	63000
110	80	2	5	4	4	100	0	256507	1026028	42949532	19000692	65520	150480	1070920	0	56760

111	80	3	5	4	4	100	0	280031	1120124	50635902	4312562	71845	164932	1227767	0	60816
112	80	3	5	4	4	100	0	267530	1070120	47191088	20613128	70960	151800	1153480	0	56720
113	80	1	5	4	4	100	0	255291	1021164	44132255	3611695	64960	147000	1062000	0	56960
114	80	1	5	4	4	100	0	262281	1049124	46613714	4749949	66078	152471	1109020	0	63765
115	80	1	5	4	4	300	7.5	247093	988372	45603486	3121947	59289	134461	1116158	38658	59197
116	80	2	5	4	4	300	7.5	252352	1009408	48895091	3615304	63333	138987	1191000	39293	56827
117	80	1	3	3	3	200	2.5	305043	915129	41929330	3109827	58372	129030	1013518	37613	48090
118	80	1	5	4	4	300	7.5	243174	972696	45375838	3421343	61413	133030	1093426	36145	54733
119	80	1	7	5	5	200	2.5	201451	1007255	48437484	2709142	67302	143688	1215767	54851	63045
120	80	1	7	3	3	200	2.5	338113	1014339	48644955	3432723	64005	138754	1169734	41291	57768
121	80	2	3	5	5	200	2.5	197395	986975	46336568	20447893	62450	134325	1108725	34625	57850
122	80	2	7	3	3	200	2.5	335179	1005537	47460421	3723376	62683	138905	1145005	44216	64372
123	80	2	3	3	3	200	2.5	337675	1013025	45713931	3480916	63179	139284	1106104	35925	54388
124	80	2	7	5	5	200	2.5	202669	1013345	47766804	21211904	62562	137811	1156791	39677	56318
125	80	3	3	3	3	200	2.5	337649	1012947	47910574	3614441	63118	138500	1145662	40162	55132
126	80	3	7	5	5	200	2.5	210287	1051435	49473863	2961913	66825	149525	1221025	43250	61825
127	80	3	3	5	5	200	2.5	206673	1033365	56469788	9725141	66187	145000	1175429	41793	55429
128	80	3	7	3	3	200	2.5	351837	1055511	51272881	3270403	67090	152179	1255000	43716	62433
129	80	1	3	5	5	200	2.5	310565	1552825	70022206	4176738	99286	221847	1708818	61527	85025

130	80	1	7	3	3	200	5	340634	1021902	47166501	3008090	65183	145530	1165515	38406	58619
131	80	1	3	3	3	200	5	308962	926886	41705681	2579178	56365	132133	1018049	33887	50261
132	80	1	3	5	5	200	5	197010	985050	46047619	3894847	64010	143292	1116411	35792	56213
133	80	2	3	3	3	200	5	333639	1000917	46678621	3353427	65985	138358	1133433	46731	61090
134	80	2	7	5	5	200	5	202388	1011940	47601103	4132736	60452	139447	1115352	37010	55804
135	80	2	3	5	5	200	5	202162	1010810	48071854	3928785	66361	137847	1132599	37153	55718
136	80	2	7	3	3	200	5	338582	1015746	46513120	3205344	60284	137403	1122731	37642	58657
137	80	3	3	5	5	200	5	204851	1024255	48045622	3662886	63706	140920	1140547	40995	57687
138	80	3	7	3	3	200	5	351105	1053315	49489749	3539005	62442	144437	1187472	39151	57000
139	80	3	3	3	3	200	5	352571	1057713	49246262	3085082	64335	148110	1204080	34230	56940
140	80	3	7	5	5	200	5	319377	1596885	77914449	5774643	97330	218519	1852913	70388	91044
141	80	2	5	4	4	300	7.5	247948	991792	49852589	3756283	64080	141400	1211520	43093	65187
142	80	3	5	4	4	300	7.5	233287	933148	46094039	3500589	57181	129933	1087315	43356	57195
143	80	3	5	4	4	300	7.5	232276	929104	46624471	3470823	73661	136133	1131894	42870	61050
144	80	1	7	5	5	200	5	199361	996805	47481799	20560537	65545	134851	1156683	37847	57376
145	80	1	5	4	4	300	0	341414	1365656	63623285	4577653	81284	186970	1539746	46020	75599
146	80	1	5	4	4	300	0	249682	998728	45732105	2674316	60316	135697	1121908	38434	57868
147	80	1	5	4	4	300	0	248608	994432	47004152	3240852	61003	134865	1153980	42587	60198
148	80	1	1	4	4	300	0	28861	115444	8890540	1258460	0	73316	165900	0	0

149	80	1	9	4	4	300	0	263393	1053572	56409257	4127799	64589	123304	1304923	46288	66381
150	80	1	5	4	4	300	0	253015	1012060	46888076	3188276	62187	137493	1160587	38507	54907
151	80	1	5	4	4	300	0	250308	1001232	46512877	2746904	62539	134487	1134566	41395	60355
152	80	1	5	4	4	300	0	260179	1040716	50125703	3445558	67355	145474	1204026	37566	57329
153	80	1	5	2	2	300	0	430171	860342	39162664	2688137	53213	126167	949047	31260	47827
154	80	1	5	6	6	300	0	168840	1013040	50173510	5232250	63440	136060	1152420	42760	59440
155	80	1	5	4	4	300	0	142202	568808	27812539	11975645	38093	114173	611613	22387	32600
156	80	1	5	4	4	300	0	142078	568312	28005358	2454918	37133	114267	610280	22107	35040
157	80	1	1	4	4	300	0	26263	105052	8865824	1731871	0	73262	154724	0	0
158	80	1	9	4	4	300	0	260351	1041404	50606762	3719886	64261	120105	1209686	41987	65268
159	80	2	5	4	4	300	0	253472	1013888	47615669	2836146	63113	138132	1176013	39709	54305
160	80	2	5	4	4	300	0	256604	1026416	49650838	3631694	66167	146301	1217124	43130	59452
161	80	2	5	4	4	300	0	252614	1010456	47401463	2923676	61200	136787	1157173	37787	55453
162	80	1	5	2	2	300	0	424422	848844	39101461	2601283	53287	122673	956957	31122	47175
163	80	1	5	6	6	300	0	166033	996198	46752108	3457525	63915	135541	1108662	38774	55318
164	80	2	5	4	4	300	0	253391	1013564	48719299	3201483	60855	140789	1179026	40592	61066
165	80	2	1	4	4	300	0	53617	214468	14054500	1728148	21196	77595	267402	0	19003
166	80	2	9	4	4	300	0	238733	954932	49861945	3703094	58139	131393	1193201	40977	62139
167	80	2	5	4	4	300	0	241643	966572	46239313	3383549	58144	127935	1104026	39856	52418

168	80	2	5	4	4	300	0	233029	932116	46341906	3356104	58344	129020	1104795	39642	59868
169	80	2	5	4	4	300	0	241908	967632	47530838	3502798	59507	129840	1136840	37187	58013
170	80	2	5	2	2	300	0	465860	931720	44312055	3000731	54890	127110	1079284	35284	51077
171	80	2	5	6	6	300	0	157793	946758	47414476	3832021	72742	128609	1111104	36140	56448
172	80	2	5	4	4	300	0	238248	952992	48257774	3284767	60027	131423	1163718	39141	51329
173	80	2	1	4	4	300	0	53651	214604	14748419	2626166	20387	82773	262520	0	16813
174	80	2	9	4	4	300	0	245146	980584	49134268	3113048	60682	134754	1179187	39043	60774
175	80	3	5	4	4	300	0	236035	944140	45974485	2957774	58577	130228	1103463	36268	55745
176	80	3	5	4	4	300	0	237767	951068	47390520	3577441	61961	128526	1127434	40303	60697
177	80	3	5	4	4	300	0	236823	947292	45501259	2772821	58278	130755	1095589	39841	57391
178	80	2	5	2	2	300	0	474614	949228	44806658	2978638	56747	126492	1097111	34714	52047
179	80	2	5	6	6	300	0	184996	1109976	55340547	3902467	67711	154691	1326161	54181	70027
180	80	3	5	4	4	300	0	237386	949544	47571049	3692889	59640	130880	1133707	40000	62000
181	80	3	1	4	4	300	0	74938	299752	18615081	2479335	24147	85097	359010	0	22555
182	80	3	9	4	4	300	0	242482	969928	48099004	3758467	58617	134685	1128215	36349	54564
183	80	3	5	4	4	300	0	242256	969024	46972200	3743256	56813	130400	1120236	33115	53587
184	80	3	5	4	4	300	0	237211	948844	46472750	3406993	59738	128800	1115384	36525	53744
185	80	3	5	4	4	300	0	236004	944016	45627711	2837031	60000	129254	1102812	42323	64277
186	80	3	5	2	2	300	0	464067	928134	44402797	2989479	54749	127425	1090314	36388	53987

187	80	3	5	6	6	300	0	155121	930726	47456253	21552139	62980	135201	1107544	44477	55510
188	80	3	5	4	4	300	0	237210	948840	46395252	3404788	59377	127510	1111947	34848	53974
189	80	3	1	4	4	300	0	79539	318156	18751349	2050557	24617	87020	379248	0	23960
190	80	3	9	4	4	300	0	239390	957560	46950496	3282193	59263	131882	1118066	40158	53079
191	80	1	5	4	4	300	0	242001	968004	44699701	2777406	60872	131718	1083812	35060	55007
192	80	2	3	3	3	400	2.5	287109	861327	41850318	3271585	52860	121043	999855	30953	47910
193	80	3	5	2	2	300	0	467041	934082	44695390	2967804	55244	127645	1098388	36328	53799
194	80	3	3	5	5	400	2.5	188546	942730	48018146	2959904	58853	129476	1149875	35873	54414
195	80	3	5	6	6	300	0	151288	907728	46347766	3453131	61192	133490	1117669	38483	53702
196	80	1	3	5	5	400	2.5	172441	862205	41067678	2494282	53097	115137	974527	33333	46741
197	80	1	3	5	5	400	2.5	170790	853950	40719859	2402533	52923	114092	968209	34328	49154
198	80	1	3	5	5	400	2.5	170151	850755	40805728	2529049	53756	116381	967363	34440	55012
199	80	1	7	3	3	400	2.5	311114	933342	45388030	2918332	53761	126045	1113359	37496	54516
200	80	1	3	3	3	400	2.5	214431	643293	30866341	2526027	41410	104612	701463	27978	38104
201	80	1	7	5	5	400	2.5	192233	961165	49722215	3711478	59325	128625	1177063	37313	58288
202	80	2	7	5	5	400	2.5	187780	938900	48473666	3695324	65773	131434	1145786	36446	59676
203	80	2	3	5	5	400	2.5	186282	931410	44610120	2763378	56082	123072	1066692	40249	60025
204	80	2	7	3	3	400	2.5	331339	994017	49633410	3403024	58730	134168	1197988	39260	57616
205	80	2	3	5	5	400	5	184056	920280	46430444	2839327	62072	127878	1106030	34020	54491

206	80	3	7	5	5	400	2.5	191564	957820	48597060	3519808	60784	133955	1164540	40100	59701
207	80	3	7	3	3	400	2.5	316427	949281	47900823	3383717	58379	132256	1160631	38374	56148
208	80	3	3	3	3	400	2.5	304651	913953	43932559	3081722	53585	123778	1068393	33941	51252
209	80	3	7	5	5	400	5	188026	940130	47599226	3045980	59055	131244	1149726	41206	55585
210	80	1	3	3	3	400	5	213413	640239	32396021	2926245	42097	105694	735560	22246	36978
211	80	1	7	5	5	400	5	190517	952585	48416524	3507574	60877	128049	1140926	44037	58383
212	80	1	3	5	5	400	5	166774	833870	41488701	2850639	54288	119363	990750	30763	50163
213	80	1	7	3	3	400	5	300197	900591	43755682	2899726	55111	124941	1086096	33585	50919
214	80	2	7	3	3	400	5	324458	973374	48643109	3402818	57030	132539	1193424	37257	59626
215	80	2	3	3	3	400	5	281383	844149	40913046	2872398	51514	119799	985846	31377	52861
216	80	2	7	5	5	400	5	194056	970280	50778506	4244126	61055	136203	1183660	39888	55881
217	80	3	3	3	3	400	5	304948	914844	44199087	2846350	56639	125895	1083669	34767	53714
218	80	3	3	5	5	400	5	186728	933640	47152614	3068419	59183	126634	1135804	37797	51559
219	80	3	7	3	3	400	5	321830	965490	48281777	3129531	59487	133862	1197565	37485	57317
220	80	1	5	4	4	500	0	232217	928868	45063200	3411640	54176	125232	1080608	36024	51288
221	80	1	5	4	4	500	0	220197	880788	43887309	2750628	54186	122273	1061735	34535	53180
222	80	2	5	4	4	500	0	242902	971608	48858700	3190149	59329	135633	1178890	39170	58555
223	80	2	5	4	4	500	0	243382	973528	49002869	3323820	59184	131081	1188372	38891	56863
224	80	3	5	4	4	500	0	233614	934456	46568638	2942449	56481	127840	1130741	36128	60641

225	80	3	5	4	4	500	0	235206	940824	47167340	3010489	57542	128056	1156691	38056	56466
226	100	2	5	4	4	100	0	232448	929792	41865115	2714422	53941	139050	1016752	0	55683
227	100	2	5	4	4	300	7.5	234027	936108	46576420	20640048	57037	143960	1102897	44173	59721
228	100	3	5	4	4	300	7.5	231889	927556	44384937	19487343	56266	131947	1068359	35854	60625
229	100	1	5	4	4	100	0	247184	988736	44092227	3682387	65760	136280	1064360	0	55560
230	100	1	5	4	4	300	7.5	351112	1404448	70097151	5267690	83684	197066	1693184	52711	82408
231	100	1	1	4	4	300	0	80397	321588	19641545	1916659	27060	89879	384980	0	22899
232	100	1	9	4	4	300	0	244755	979020	49819957	3922797	65600	142427	1184720	46387	61240
233	100	2	5	4	4	300	0	233630	934520	46391091	3545004	57659	135358	1108709	33124	59090
234	100	2	5	4	4	300	0	235407	941628	45635334	3575240	56805	129678	1091128	36940	54899
235	100	2	5	4	4	300	0	233372	933488	47073976	3922802	61390	141089	1114872	54387	77233
236	100	1	3	3	3	200	2.5	317552	952656	44538187	3210860	56678	128678	1060888	38341	57146
237	100	1	5	4	4	300	0	237194	948776	45929839	3048679	56187	132560	1094213	40133	63133
238	100	2	3	5	5	200	2.5	194400	972000	47953838	20763715	60123	138892	1128473	39458	60320
239	100	2	7	3	3	200	2.5	317392	952176	44398195	19578640	59220	132300	1061130	39435	56235
240	100	3	3	3	3	200	2.5	315515	946545	44334798	3011261	57612	134134	1071179	33955	52030
241	100	3	7	5	5	200	2.5	184630	923150	42634465	18800311	59925	130274	1031418	40721	59950
242	100	1	5	4	4	300	0	231662	926648	45332030	19695618	56691	130804	1083654	35761	52173
243	100	1	7	3	3	200	5	333931	1001793	47233004	3409720	62970	136657	1140746	40761	54821

244	100	2	3	3	3	200	5	318713	956139	44242523	29	56000	131075	1059478	38791	57597
245	100	2	7	5	5	200	5	188047	940235	45616224	3595138	62955	158384	1093939	39747	72955
246	100	3	3	5	5	200	5	182378	911890	41914455	2821757	56064	127772	1010693	32104	50322
247	100	3	7	3	3	200	5	321512	964536	44477722	3825431	57136	138799	1057839	36362	50653
248	100	2	5	4	4	300	0	243773	975092	47494802	3560701	60323	137495	1128902	35677	56714
249	100	2	1	4	4	300	0	117616	470464	26266220	11051799	33118	97539	563158	19461	30342
250	100	2	9	4	4	300	0	236592	946368	48126465	21541176	62309	143557	1141315	39302	59933
251	100	3	5	4	4	300	0	238680	954720	45698119	3496066	57632	134829	1098592	38197	52263
252	100	3	5	4	4	300	0	235223	940892	46313584	3216610	58587	135253	1131627	35147	52907
253	100	3	5	4	4	300	0	233107	932428	44634998	19638300	57421	132355	1074539	33632	54434
254	100	3	5	4	4	300	0	249910	999640	49108212	21851375	65076	145648	1192106	41528	58525
255	100	3	1	4	4	300	0	139781	559124	28604309	2033352	38086	100013	634442	18970	34007
256	100	3	9	4	4	300	0	234162	936648	45578661	19996962	62941	137137	1108915	40575	57922
257	100	1	5	2	2	300	0	465459	930918	44594694	2961503	53873	125960	1099418	34769	53104
258	100	1	5	6	6	300	0	156933	941598	45533965	20330809	59059	126922	1072451	34176	53824
259	100	1	5	4	4	300	0	242439	969756	47151682	3152926	56468	134462	1152923	44923	62328
260	100	1	5	4	4	300	0	237807	951228	47052575	3605083	57191	131144	1133632	49084	56013
261	100	2	5	2	2	300	0	469324	938648	44431981	3220737	56293	129967	1088046	35303	54274
262	100	2	5	6	6	300	0	165686	994116	43981524	19279604	61010	128178	1067624	43644	58871

263	100	1	7	5	5	200	2.5	190753	953765	46859717	3569767	61625	140850	1130000	38100	58825
264	100	1	3	5	5	200	5	199392	996960	46455489	3318464	59000	133750	1098550	58175	59725
265	100	3	5	2	2	300	0	574678	1149356	55059044	3922361	68769	163291	1360247	44629	66067
266	100	3	5	6	6	300	0	153246	919476	44900486	19662598	59702	130371	1081470	32682	56364
267	100	1	3	5	5	400	2.5	187794	938970	47530751	3196559	59341	139590	1152264	43756	64838
268	100	1	7	3	3	400	2.5	328724	986172	49088322	3333485	59948	136526	1208652	39881	58126
269	100	2	3	3	3	400	2.5	306806	920418	45634001	3159279	56707	126173	1109902	36271	54940
270	100	2	7	5	5	400	2.5	197208	986040	48378880	3565922	65238	141103	1166416	40689	59724
271	100	3	3	5	5	400	2.5	181647	908235	45429300	2888567	58868	129726	1100920	40933	59714
272	100	3	7	3	3	400	2.5	315038	945114	46059194	3247299	58293	135932	1132263	35150	57985
273	100	1	3	3	3	400	5	314023	942069	45904194	3316632	55455	129394	1106948	37507	55842
274	100	1	7	5	5	400	5	193504	967520	49432058	3725153	58258	135714	1167293	43797	61817
275	100	2	3	5	5	400	5	187852	939260	48343586	3515169	57544	133017	1169701	42905	64888
276	100	2	7	3	3	400	5	322056	966168	48130793	3475904	61975	138349	1172257	38941	57646
277	100	3	3	3	3	400	5	332537	997611	49106979	3700230	62434	138841	1207824	39893	61027
278	100	3	7	5	5	400	5	172272	861360	39480962	3139395	55501	133070	986441	32318	47306
279	100	2	5	4	4	500	0	232735	930940	46092695	3304591	57339	128223	1119291	36143	58032
280	100	3	5	4	4	500	0	237315	949260	46524698	3217726	58829	132303	1145211	38000	54127

Table 1 continuation

TP	TM	CO	VT	VA	M	H	C14:1	C15:0	iso C16	C16:0	iso C17	C16:1	anteiso C17	C17:0	C17:1	C18:0	C18:1 9t
60	1	5	4	4	100	0	0	106360	0	7822040	141200	378320	145440	335760	178640	5379200	70320
60	3	5	4	4	100	0	0	123683	0	9619129	145465	455762	160594	410495	216673	6675723	87683
60	1	3	3	3	200	2.5	0	72075	23104	5396090	103239	244209	106552	220164	120776	3634627	46448
60	1	7	5	5	200	2.5	0	106863	0	8347451	144534	389216	152917	344657	188333	5730000	69877
60	2	3	5	5	200	2.5	0	108867	0	8554631	142118	388300	153005	352438	188695	5906158	68892
60	3	3	3	3	200	2.5	27161	112039	22449	8625995	131137	390205	146605	350473	180337	5951971	74780
60	3	7	5	5	200	2.5	0	137075	0	10665300	171125	489600	184400	441775	226925	7442000	95100
60	1	3	5	5	200	5	0	81122	0	6136000	106220	286927	119610	257049	136000	4208634	51512
60	1	7	3	3	200	5	18761	87176	0	6857151	117659	321527	120820	281561	149429	4652795	59854
60	2	7	5	5	200	5	31269	122214	0	9579279	150498	442239	167886	392985	209229	6648134	87065
60	3	3	5	5	200	5	0	120735	0	9524755	146887	436446	183186	399240	215858	6571691	78873
60	3	7	3	3	200	5	27790	123863	24527	9886566	157522	442771	180541	399029	212941	6873380	87951
60	1	5	4	4	300	7.5	18676	79371	21592	6273793	99625	280348	103037	252749	141472	4293110	53110
60	2	5	4	4	300	7.5	24647	109017	20634	8749122	137175	386178	148739	350772	189518	6088092	75366
60	3	5	4	4	300	7.5	26724	123618	24895	9967132	157382	439803	186803	398974	210026	6951355	95645
60	1	5	4	4	300	0	20226	94365	21515	7289635	118618	328080	132372	296120	162512	4987522	63056
60	1	5	4	4	300	0	19147	86493	23027	6903973	114107	309667	117573	278747	154827	4728013	61587
60	1	5	4	4	300	0	23303	86855	20289	6936000	111895	310105	116316	279408	154421	4756645	66697
60	1	5	4	4	300	0	18475	89431	21779	6867612	114355	310676	125726	277819	155518	4707946	54221
60	1	1	4	4	300	0	0	55010	20066	4373109	74086	189690	77914	179987	102640	2978548	37927
60	1	9	4	4	300	0	0	63087	25329	4904336	83933	238913	83463	201342	127128	3288349	38013
60	1	5	2	2	300	0	11658	54168	19322	4393651	73188	193282	80973	174248	100383	2970953	35711
60	1	5	6	6	300	0	0	97822	0	7539941	125267	348851	137248	312436	170733	5198119	64554
60	2	5	4	4	300	0	28161	117987	17732	9418000	154685	425839	158081	380000	205718	6523195	77450
60	2	5	4	4	300	0	27314	120040	27696	9606284	157096	428977	175287	382772	205175	6664594	80211

60	2	5	4	4	300	0	26361	120052	24197	9267882	143541	416459	164315	371685	195830	6418007	84511
60	2	1	4	4	300	0	24915	88654	19582	7036026	115255	315438	122301	281961	152118	4822092	56680
60	2	9	4	4	300	0	20354	88157	21154	6986177	115030	319633	121652	280748	162951	4760800	55318
60	2	5	6	6	300	0	27632	123257	0	9848309	153671	445638	163243	422092	221211	6816711	80586
60	3	5	4	4	300	0	29325	133272	26203	10385443	170649	468656	197810	417757	224498	7262361	89036
60	3	5	4	4	300	0	29067	127320	26293	10094893	154507	454107	193320	402413	212800	6983773	81627
60	3	5	4	4	300	0	28174	126013	20604	9978322	151275	440309	179611	395329	217342	6957248	83087
60	3	5	4	4	300	0	33342	126376	31987	10212134	157128	461691	184859	407530	217691	7102443	94201
60	3	1	4	4	300	0	23086	103325	23483	8315430	132199	369007	148464	332517	177391	5731722	65258
60	3	9	4	4	300	0	25450	111369	31960	8908832	142054	399289	155839	354309	193342	6177557	75369
60	3	5	2	2	300	0	25638	105463	23537	8530758	133926	375738	152624	335852	180342	5919356	73067
60	3	5	6	6	300	0	29467	138020	0	11043789	169342	490934	175875	456711	231553	7674039	98467
60	1	3	5	5	400	2.5	0	65000	23816	5113753	87065	223741	94093	209433	117128	3496398	44761
60	1	7	3	3	400	2.5	15119	64515	20406	5245552	86309	232975	91604	205374	122101	3554079	39876
60	2	3	3	3	400	2.5	13319	62541	20437	5081874	82970	222600	92519	205978	116800	3460274	38837
60	3	3	5	5	400	2.5	26858	112805	22818	8721870	137406	388005	150461	351933	185923	6015499	80125
60	3	7	3	3	400	2.5	26752	116654	23023	9563827	148045	422015	169143	377820	201549	6683038	78805
60	1	3	3	3	400	5	0	38341	21242	3016174	56091	129508	49356	120856	71492	1995492	24386
60	1	7	5	5	400	5	19799	89636	21822	7157399	115075	320829	129472	286922	159761	4899246	58819
60	2	3	5	5	400	5	19621	86869	21439	7077588	118371	316629	137083	282462	162109	4854419	58131
60	2	7	3	3	400	5	20087	91212	22309	7364155	117756	333905	121825	292362	163279	5059953	56431
60	3	3	3	3	400	5	16615	73348	19570	6026896	97822	262007	109119	239400	132281	4140030	49578
60	3	7	5	5	400	5	30126	129497	21269	10516256	171319	474146	178480	435138	222776	7384347	95704
60	1	5	4	4	500	0	16633	74461	21932	6026597	100721	268938	109050	243663	136393	4136770	46541
60	2	5	4	4	500	0	23197	105205	20120	8544185	133952	383149	150169	341904	181574	5944827	66675
60	3	5	4	4	500	0	34733	134108	30244	10778934	158741	470549	172954	420954	225587	7512609	91142
70	1	5	4	4	100	0	0	119612	0	9135689	111184	424388	152466	389748	208816	6386019	83495
70	1	3	5	5	200	2.5	0	110025	37525	8581500	102300	394275	154050	354375	190200	5916825	75650
70	1	7	5	5	200	5	31616	124268	43485	9756818	114571	444369	175354	400126	215631	6785126	84545

70	2	5	2	2	300	0	26772	119472	41294	9679941	111327	429234	175512	384733	200990	6821413	80700
70	1	5	4	4	300	7.5	23933	101745	34591	8151436	94899	363101	159987	329960	176698	5637423	67034
70	1	7	3	3	200	2.5	28568	117467	41095	9386804	108060	423045	177859	376568	203397	6499628	80533
70	2	3	3	3	200	2.5	27912	119176	40853	9541162	110221	425265	174956	382544	201971	6658044	81368
70	2	7	5	5	200	2.5	32675	136925	50250	10769675	124925	483675	195200	442325	233750	7566750	97075
70	3	7	3	3	200	2.5	29925	130254	47224	10478418	119776	464970	188776	421045	221403	7398597	91179
70	1	3	3	3	200	5	20367	86307	28417	6934598	82251	311261	130070	280402	156241	4781382	59111
70	1	5	4	4	300	0	26462	115398	39010	9141378	105338	407505	154662	365137	194234	6360950	78100
70	2	3	5	5	200	5	31915	127264	45547	10173806	120124	464502	181194	419030	220995	7078209	82836
70	2	7	3	3	200	5	31379	131030	46803	10626455	120879	476197	180030	427439	225000	7465985	91545
70	3	3	3	3	200	5	28856	125376	42460	9949604	114401	451782	167376	399000	210446	6956139	86064
70	3	5	2	2	300	0	28530	123711	43537	10003651	114893	445826	172913	395819	206483	7078148	87242
70	3	3	5	5	200	2.5	0	126616	43409	9910278	118308	462904	176364	408889	216540	6887146	88737
70	3	7	5	5	200	5	33100	136550	47700	10766475	126200	492600	191500	439050	230675	7531425	97325
70	1	5	4	4	300	0	26747	113480	40053	9150520	104747	408413	171640	364907	194893	6361307	77440
70	2	5	4	4	100	0	0	128431	0	9597137	115412	449020	177882	407333	213098	6698706	91137
70	3	5	4	4	100	0	0	131269	48692	9736154	119846	462038	180692	415577	216077	6918692	85962
70	1	5	2	2	300	0	17848	79413	27162	6516964	74205	289050	113010	256040	141974	4502106	54944
70	1	5	6	6	300	0	28579	125783	44467	9891928	113941	442599	172875	403421	213079	6890625	87671
70	1	5	4	4	300	0	26033	116380	40092	9298570	109744	406911	163161	374859	198072	6463449	75987
70	1	5	4	4	300	0	27037	117712	40575	9476361	107906	418943	182930	378569	202435	6590783	79906
70	1	7	5	5	400	2.5	28913	125200	44813	10199250	116400	449938	178463	406688	215975	7154288	81038
70	1	3	5	5	400	5	19454	87928	30136	7097916	83387	313164	134814	284355	155335	4898697	61625
70	1	1	4	4	300	0	0	0	0	1105409	22339	51136	17116	47083	27787	686365	0
70	1	9	4	4	300	0	24640	111427	39920	8884467	102587	403120	154853	355707	194800	6161813	68120
70	2	5	4	4	300	7.5	30046	124892	43725	10128118	115423	453377	173692	404157	214046	7074138	81954
70	3	5	4	4	300	7.5	30733	129254	45545	10513122	119102	475116	174257	419248	223894	7384251	85637
70	2	5	4	4	300	0	29528	125688	45369	10176611	116120	455163	176292	408784	217050	7131774	87814
70	2	5	4	4	300	0	29333	129693	45253	10495493	118880	464200	183627	420373	221160	7363773	89147

70	2	5	4	4	300	0	30954	127854	44490	10332040	121205	456755	201113	411947	218781	7228821	89351
70	2	5	4	4	300	0	30199	130649	46159	10608967	120146	467245	186252	423245	225947	7438967	89748
70	2	1	4	4	300	0	0	20053	0	1459208	18904	66997	22878	61848	42944	926799	0
70	2	9	4	4	300	0	30026	127603	44411	10357589	117682	458636	180305	415695	220053	7298199	88384
70	3	5	4	4	300	0	29603	127444	46556	10377377	118159	460583	178543	414795	219020	7289523	91775
70	3	5	4	4	300	0	30544	132616	48734	10663698	123934	474820	179711	424734	225862	7517731	86925
70	1	3	3	3	400	2.5	12720	59153	19181	4796629	56124	205864	88931	191027	108498	3267876	39208
70	1	5	4	4	500	0	22317	98028	33331	7947663	90501	348016	145074	316289	169643	5522862	65611
70	2	3	5	5	400	2.5	27010	122261	43957	9917563	113229	433631	170917	398656	208794	6977525	80302
70	2	7	3	3	400	2.5	28107	122776	44229	10016791	111314	443237	178167	398379	208534	7092688	77020
70	3	3	3	3	400	2.5	24075	105246	36716	8563933	96149	379896	139164	336896	179015	5977463	70828
70	3	7	5	5	400	2.5	32196	135819	50608	11146799	126464	490645	191303	445422	232419	7903313	95695
70	1	7	3	3	400	5	21292	97010	33082	7838618	88923	344200	145586	308319	169145	5437691	65978
70	2	3	3	3	400	5	18299	85840	29027	6936569	79728	304272	121601	274302	149431	4825070	57030
70	2	7	5	5	400	5	30373	136468	48532	11005634	125348	485062	191803	440373	230224	7785659	90211
70	3	3	5	5	400	5	29350	125700	43325	10164875	114125	453275	170563	404488	213600	7096250	86025
70	3	7	3	3	400	5	29291	128328	46799	10541716	116806	465769	175373	416433	218940	7469806	85903
70	3	5	4	4	300	0	30631	128425	46100	10452412	118299	469342	176678	416983	219096	7349542	90272
70	3	5	4	4	300	0	30980	134768	49616	10854185	123603	482437	185073	435497	228384	7661722	88543
70	3	9	4	4	300	0	33453	145573	53760	11846920	134147	521960	213747	472973	248227	8374253	100693
70	2	5	4	4	500	0	28952	125736	44864	10365448	116104	462200	181384	411256	216048	7305624	88488
70	3	1	4	4	300	0	0	26671	0	2079275	27597	93302	34094	88268	56805	1355356	18913
70	3	5	4	4	500	0	29451	128000	46605	10581315	118669	468297	189547	419471	218485	7498333	92689
70	2	5	6	6	300	0	30098	130092	47036	10371816	120315	464715	181948	421652	220997	7273515	92066
70	3	5	6	6	300	0	31574	138059	49889	10980354	131134	483836	193613	446577	231148	7736144	98341
80	2	5	4	4	100	0	0	137800	0	10217920	130520	467600	176360	429800	203080	7160600	92560
80	2	5	4	4	100	0	0	129880	0	9754120	111480	162760	0	418000	225560	6849480	86400
80	3	5	4	4	100	0	0	145087	50563	11027029	138019	531534	210641	477553	247301	7827961	102058
80	3	5	4	4	100	0	0	139840	0	10535640	123840	485680	185240	445720	231560	7411800	97800

80	1	5	4	4	100	0	0	125240	0	9676440	113080	445560	155960	416360	216480	6732080	93760
80	1	5	4	4	100	0	0	143569	65608	10035686	110000	453686	158627	418784	216549	6960824	91725
80	1	5	4	4	300	7.5	31776	125618	50368	10190526	115632	447618	169118	406526	213342	7133829	85776
80	2	5	4	4	300	7.5	30933	134267	52000	10919440	127320	474360	201027	439813	225560	7732560	91520
80	1	3	3	3	200	2.5	26910	115749	41095	9375844	107126	412658	165980	376040	200970	6522045	83020
80	1	5	4	4	300	7.5	28317	127894	44541	10077980	114389	451459	180581	402178	209373	7049254	87564
80	1	7	5	5	200	2.5	36460	140545	50916	10962054	131139	503391	185198	457748	244678	7657376	95446
80	1	7	3	3	200	2.5	32897	135739	46951	10869680	123325	489635	182645	434202	224837	7634379	88448
80	2	3	5	5	200	2.5	0	129225	43125	10320525	121050	473675	183875	430850	217500	7228975	82850
80	2	7	3	3	200	2.5	31553	132045	48784	10498990	119397	469945	183739	428487	227261	7424337	93000
80	2	3	3	3	200	2.5	30090	125313	43687	10187537	117821	462239	191806	403493	214776	7082955	84194
80	2	7	5	5	200	2.5	31368	137065	52488	10559900	128433	484254	192214	437637	228607	7399453	87164
80	3	3	3	3	200	2.5	28838	130471	50132	10615294	121500	467235	208191	423132	223926	7477279	100015
80	3	7	5	5	200	2.5	36450	142375	55875	11132700	130800	530025	211125	470700	247075	7861575	105050
80	3	3	5	5	200	2.5	33687	141212	49470	10731061	126439	496389	193914	452197	236465	7581641	102626
80	3	7	3	3	200	2.5	36701	144716	49537	11433910	133313	522866	205642	469701	245045	8144970	97761
80	1	3	5	5	200	2.5	48621	191429	75739	15876946	179039	709360	265345	633719	336305	11018374	128941
80	1	7	3	3	200	5	31351	134391	45698	10501663	125970	490485	184604	439262	232990	7471931	90327
80	1	3	3	3	200	5	28123	117340	38734	9463626	105798	422601	158704	377158	197350	6568300	78709
80	1	3	5	5	200	5	31658	131757	48267	10035297	118391	473985	183787	420891	226757	7025520	89356
80	2	3	3	3	200	5	31806	127478	46940	10452254	118567	467493	178925	415821	217597	7301030	90522
80	2	7	5	5	200	5	32915	133719	52010	10375025	121055	478769	188317	426608	227136	7268467	89497
80	2	3	5	5	200	5	0	134827	44356	10604183	118713	474307	192995	434554	226584	7432475	92475
80	2	7	3	3	200	5	30761	126343	46866	10424925	117045	462000	183776	418299	219642	7330194	86224
80	3	3	5	5	200	5	33756	132313	46095	10630920	123930	487239	188035	432114	231791	7427562	93060
80	3	7	3	3	200	5	32487	133839	44322	11031121	125322	486271	210829	438965	229975	7778231	101744
80	3	3	3	3	200	5	31590	136890	50820	11006895	128100	497700	195435	451245	234465	7806600	100635
80	3	7	5	5	200	5	52063	207549	84272	17399587	193155	761262	294782	686578	355049	12237961	157354
80	2	5	4	4	300	7.5	32227	140387	48813	11101133	130507	484507	199667	455093	232840	7927853	97453

80	3	5	4	4	300	7.5	32228	122148	44456	10233450	115423	455826	189705	404107	209597	7209154	87919
80	3	5	4	4	300	7.5	33076	125143	45621	10505967	116904	456199	176917	410419	207136	7348359	90392
80	1	7	5	5	200	5	31955	135941	55074	10706881	131510	490074	199134	447030	225990	7493639	91807
80	1	5	4	4	300	0	39826	175318	65191	14184254	157793	621726	232870	564990	293391	10002261	117860
80	1	5	4	4	300	0	31474	128197	45724	10340974	117579	465697	178224	412197	216276	7232303	92408
80	1	5	4	4	300	0	31894	133690	49386	10509571	120185	463617	195406	423472	221716	7373861	92766
80	1	1	4	4	300	0	0	23721	0	1846179	21382	83668	29914	80465	50631	1197940	0
80	1	9	4	4	300	0	35505	148147	59920	12487893	139090	539264	209445	499612	260094	9037017	115077
80	1	5	4	4	300	0	30827	132600	40800	10510667	118960	463973	179080	420053	217453	7380840	90293
80	1	5	4	4	300	0	30039	130553	45539	10527684	117487	472566	175039	417763	220013	7373118	94316
80	1	5	4	4	300	0	32684	137711	50487	11186618	126974	495961	202250	448987	233408	7871684	96039
80	1	5	2	2	300	0	26540	109213	36393	8760273	98793	386953	156540	348200	183587	6112593	70927
80	1	5	6	6	300	0	27600	140820	49580	10772260	123660	484660	179800	438660	228340	7543820	95800
80	1	5	4	4	300	0	17160	76520	24320	6120707	104827	107507	0	243813	31853	4201613	56400
80	1	5	4	4	300	0	17187	72827	26480	6079680	73000	269733	102320	249627	138840	4188613	47093
80	1	1	4	4	300	0	0	23987	0	1738392	20811	74299	25037	80213	38060	1120532	0
80	1	9	4	4	300	0	35046	137268	50941	11258876	130327	497255	194993	450758	232196	7976183	96235
80	2	5	4	4	300	0	30583	136344	51444	10760728	120066	470583	214424	429113	221166	7587497	88702
80	2	5	4	4	300	0	32482	140575	51144	10962876	128361	512843	191813	453512	239278	7789793	92763
80	2	5	4	4	300	0	31053	130440	49880	10648787	120507	469467	183867	425640	225293	7502493	91920
80	1	5	2	2	300	0	23776	107155	37149	8777644	99875	385591	152178	346528	182515	6166515	72013
80	1	5	6	6	300	0	30866	130230	41508	10387436	120866	471895	196957	422144	215843	7248866	93030
80	2	5	4	4	300	0	31947	133316	53250	10900697	122539	488921	188092	436000	230013	7686961	94987
80	2	1	4	4	300	0	0	37714	0	2982379	35522	134206	44837	122764	78472	1968159	31203
80	2	9	4	4	300	0	33927	134693	56026	11133122	128330	480911	191287	446785	225254	7888224	86244
80	2	5	4	4	300	0	31046	122157	44915	10336379	114105	452261	174680	410967	212092	7262026	92144
80	2	5	4	4	300	0	30940	126305	44954	10348874	116940	455470	183139	407695	217510	7281046	85020
80	2	5	4	4	300	0	31813	128800	47373	10603813	117680	466973	182000	415440	216800	7455453	87760
80	2	5	2	2	300	0	29465	118247	42147	9931686	108441	430856	176087	382107	198689	7000957	84522

80	2	5	6	6	300	0	32528	133706	47197	10502528	123813	472033	182829	429291	222000	7363866	86167
80	2	5	4	4	300	0	29154	129973	50805	10850255	124779	476805	181436	426537	216268	7633154	91691
80	2	1	4	4	300	0	0	36387	0	2917413	40120	141867	52627	126173	74013	1936667	29973
80	2	9	4	4	300	0	31161	134085	53810	11080892	124498	484485	187764	444026	230374	7845652	94990
80	3	5	4	4	300	0	34242	123517	46617	10388282	115772	462376	179879	411919	215168	7275570	84161
80	3	5	4	4	300	0	32316	127737	57579	10566145	117500	456145	175224	409645	215211	7438447	90592
80	3	5	4	4	300	0	27974	124490	45642	10294636	112013	447099	185258	403563	208848	7237801	91046
80	2	5	2	2	300	0	27818	120640	43407	10037057	110505	442263	197697	405071	202519	7074761	90485
80	2	5	6	6	300	0	40872	157067	49933	12424027	143154	550792	214651	495040	253087	8717376	101758
80	3	5	4	4	300	0	33947	129240	45987	10575427	118373	460027	179147	415773	217800	7452067	100360
80	3	1	4	4	300	0	0	51371	17525	3897217	47679	171946	63452	163559	88789	2618127	39973
80	3	9	4	4	300	0	31141	126497	50510	10662993	119181	474604	177906	422577	219839	7541718	106980
80	3	5	4	4	300	0	29797	125233	44708	10408210	121902	469390	186505	424944	216144	7329626	85456
80	3	5	4	4	300	0	32407	126754	49797	10379069	115974	466413	175462	411646	211921	7356905	87777
80	3	5	4	4	300	0	34917	123868	47789	10328422	114482	451710	187987	406310	214904	7270865	86548
80	3	5	2	2	300	0	30829	121920	46080	9939064	109786	433786	192522	391592	198829	7040528	83338
80	3	5	6	6	300	0	37107	128799	42745	10355477	122718	471544	186725	418107	211631	7253295	95879
80	3	5	4	4	300	0	32768	125113	50000	10372384	112927	454066	173921	410570	211616	7274861	91179
80	3	1	4	4	300	0	0	54456	0	4038658	50013	179007	76577	168752	97047	2721893	38819
80	3	9	4	4	300	0	29553	125355	49211	10526184	118908	462763	178816	415421	214711	7410553	93118
80	1	5	4	4	300	0	27436	125409	44067	10035651	115356	446899	177477	405221	212940	7039396	93208
80	2	3	3	3	400	2.5	25725	112373	42548	9315173	102060	407693	147315	361245	188415	6518955	76403
80	3	5	2	2	300	0	28990	121124	43799	10045278	108569	438983	159753	390789	201010	7121485	84127
80	3	3	5	5	400	2.5	31546	131359	47731	10851521	122406	472082	188828	429813	220474	7678304	93092
80	3	5	6	6	300	0	33477	125166	45338	10394106	116384	453417	179801	415709	222258	7248139	90477
80	1	3	5	5	400	2.5	29838	110249	38632	9343346	103346	403172	153483	362985	189527	6544179	79154
80	1	3	5	5	400	2.5	27289	109303	36555	9252065	101716	399415	163881	361940	191256	6461294	83694
80	1	3	5	5	400	2.5	29714	110224	37438	9208010	104366	409142	153047	369701	191169	6450485	84714
80	1	7	3	3	400	2.5	30397	120434	43204	10229289	112279	446372	164394	402471	208781	7212105	86469

80	1	3	3	3	400	2.5	19836	81351	27664	6844843	78104	294104	111821	268134	145388	4735925	59425
80	1	7	5	5	400	2.5	34175	132238	49050	11084788	120813	483363	178100	432113	228775	7817075	91988
80	2	7	5	5	400	2.5	31397	129327	47132	10755898	117419	463678	191534	420075	221958	7631883	86808
80	2	3	5	5	400	2.5	32562	120224	42774	10071555	112512	442711	170348	395448	205025	7072910	90299
80	2	7	3	3	400	2.5	30743	132408	48022	11071203	124760	487946	178032	435572	225045	7860587	96542
80	2	3	5	5	400	5	31526	125211	54057	10491873	115323	454218	171079	410533	216538	7372841	94926
80	3	7	5	5	400	2.5	33731	131057	43134	10869527	121853	478221	181517	428520	214851	7679639	100087
80	3	7	3	3	400	2.5	31364	129724	46575	10692935	116465	461925	177256	420709	213837	7617678	101028
80	3	3	3	3	400	2.5	27963	116800	45467	9830222	111363	431422	170793	390911	199933	6922541	81785
80	3	7	5	5	400	5	29366	129502	47351	10741878	120361	468968	183221	421219	216965	7588756	97935
80	1	3	3	3	400	5	18060	84918	30351	7133881	80075	307657	122060	284022	152336	4956866	63530
80	1	7	5	5	400	5	31889	126259	56309	10807284	117370	469296	175012	418543	226889	7608469	99802
80	1	3	5	5	400	5	26225	110388	40375	9348588	103000	406188	154850	370488	192900	6528300	81350
80	1	7	3	3	400	5	28844	120030	40807	9837230	107822	432081	162489	387911	202022	6952963	76541
80	2	7	3	3	400	5	31145	130818	46287	10858810	119798	470222	180232	428791	221798	7725389	89596
80	2	3	3	3	400	5	25347	108975	35270	9187087	100146	395308	167203	358548	185844	6438290	79236
80	2	7	5	5	400	5	31377	135918	50918	11212903	126216	491266	193213	448685	222333	7935050	97419
80	3	3	3	3	400	5	28812	123090	46143	9977015	108962	432586	163474	391083	200872	7026729	87429
80	3	3	5	5	400	5	29629	126547	50928	10593750	122611	459641	180507	420062	212587	7471225	102166
80	3	7	3	3	400	5	30030	132558	46952	10904940	120256	474415	179510	432490	219626	7730359	96249
80	1	5	4	4	500	0	25840	119576	40848	10041768	110720	434760	161216	390560	205456	7069952	85216
80	1	5	4	4	500	0	29774	117014	41402	9930091	108388	430851	162709	385077	199350	6985853	86915
80	2	5	4	4	500	0	30140	129261	48447	10969317	119465	480303	189341	430036	219641	7784990	101780
80	2	5	4	4	500	0	30725	129426	47778	11002970	120547	477901	184206	431968	220982	7800483	89354
80	3	5	4	4	500	0	31126	123327	45579	10498116	114950	462782	170365	410854	212473	7434060	87455
80	3	5	4	4	500	0	31285	127743	47494	10620129	122337	470867	199221	421807	213526	7540297	83398
100	2	5	4	4	100	0	0	116396	0	9364000	105109	438812	160950	406851	209703	6553624	92950
100	2	5	4	4	300	7.5	36332	124013	44492	10361515	115987	459176	162897	402498	210498	7293449	102419
100	3	5	4	4	300	7.5	35694	122339	36173	10007508	115296	441940	167947	395694	207814	7045063	108704

100	1	5	4	4	100	0	0	140040	51240	9571400	123000	453920	170880	424480	224280	6697880	128520
100	1	5	4	4	300	7.5	43829	186079	67987	15669829	170039	678263	252474	606316	324211	11106539	135474
100	1	1	4	4	300	0	0	53664	17275	4198470	57919	187544	77517	183544	104376	2838765	44282
100	1	9	4	4	300	0	39640	146200	58413	11091600	118973	484173	208000	439707	229413	7844680	111760
100	2	5	4	4	300	0	33217	128910	54207	10365084	118716	451599	177672	413632	220174	7317391	110569
100	2	5	4	4	300	0	32685	122872	43289	10193919	115664	443369	175034	402336	195315	7189020	95718
100	2	5	4	4	300	0	46662	144407	47043	10406269	119816	453534	174584	412984	214177	7349128	110767
100	1	3	3	3	200	2.5	32063	123322	50298	9940171	111527	448346	179005	401766	203634	6983971	91156
100	1	5	4	4	300	0	35347	129293	41760	10271267	108627	439573	169520	409040	217680	7224507	96333
100	2	3	5	5	200	2.5	39926	133547	47488	10657759	125616	483300	186256	452291	229384	7491970	97956
100	2	7	3	3	200	2.5	33525	119550	37845	9936960	119505	451410	168015	395550	209430	6961425	114675
100	3	3	3	3	200	2.5	32821	121463	46254	9971164	119761	444164	179627	395955	205134	6967896	106194
100	3	7	5	5	200	2.5	32040	122065	38557	9614353	109428	430124	167711	392114	204751	6693706	113881
100	1	5	4	4	300	0	29183	122751	48478	10168757	113116	446525	173276	404066	207322	7186711	93355
100	1	7	3	3	200	5	30955	129612	41970	10654299	120224	462209	201119	419045	215806	7492731	95388
100	2	3	3	3	200	5	34358	119567	47179	9979463	115970	438806	176224	401597	207896	7015791	99567
100	2	7	5	5	200	5	41692	141338	43106	10063636	111591	461742	200960	435960	234116	7075859	108359
100	3	3	5	5	200	5	33168	116906	42030	9544876	109579	427178	188416	380990	203243	6648317	105470
100	3	7	3	3	200	5	31447	121568	46432	9889658	112342	433161	173111	395533	200171	6938608	117618
100	2	5	4	4	300	0	33522	128956	48795	10631407	118842	463825	182842	419596	224377	7498141	107111
100	2	1	4	4	300	0	23882	81263	24263	5720421	66605	243434	111355	257566	127092	3930421	65026
100	2	9	4	4	300	0	35262	140242	51517	10703101	121611	465906	189208	425919	221101	7499839	108913
100	3	5	4	4	300	0	30158	124763	39895	10247645	115526	450158	196039	404921	206526	7204066	117408
100	3	5	4	4	300	0	31840	128440	47733	10509293	118453	457627	178947	415880	213307	7409187	122813
100	3	5	4	4	300	0	32553	123289	47632	10061395	111342	447513	174592	397829	204395	7099013	120895
100	3	5	4	4	300	0	33794	133063	51269	11021940	124053	481860	191402	439455	217116	7747136	97874
100	3	1	4	4	300	0	19089	83617	23802	6369254	78587	281558	104290	256700	139683	4394640	72000
100	3	9	4	4	300	0	34366	129608	51399	10327359	115085	451137	174314	411804	204562	7291072	120641
100	1	5	2	2	300	0	30963	123278	42903	10033204	110013	438334	183023	389191	199819	7124515	91164

100	1	5	6	6	300	0	33373	130882	40843	10058196	118647	454706	181255	404157	219196	7052216	99176
100	1	5	4	4	300	0	33538	131639	53672	10653030	121351	468816	186033	419371	216294	7516803	93472
100	1	5	4	4	300	0	32415	127933	42595	10464080	113378	466943	173565	419184	209953	7361365	105151
100	2	5	2	2	300	0	30736	117583	45218	9958853	113648	447752	177121	391518	200958	7053140	99661
100	2	5	6	6	300	0	32040	125782	50475	9955743	109109	450871	169663	400515	207564	6954871	104950
100	1	7	5	5	200	2.5	41100	136525	46700	10502900	120025	468800	183050	429650	222600	7354775	96550
100	1	3	5	5	200	5	48475	138675	53325	10333600	118275	478700	198100	429725	230125	7282525	89375
100	3	5	2	2	300	0	37532	153130	54094	12332870	139532	546963	217766	497786	246408	8772033	117177
100	3	5	6	6	300	0	33656	123854	44026	10141510	115132	454013	174974	412192	205947	7126907	116166
100	1	3	5	5	400	2.5	41082	132289	59042	10676903	123184	463918	217687	425896	228818	7591654	92102
100	1	7	3	3	400	2.5	32259	134556	52844	11042274	122874	480519	185074	439156	221911	7862007	92778
100	2	3	3	3	400	2.5	30338	121737	45609	10230030	111895	444391	168165	401947	206496	7255496	95850
100	2	7	5	5	400	2.5	34574	129449	51291	10826115	120226	473784	202519	433183	219248	7675213	119348
100	3	3	5	5	400	2.5	33060	127724	48532	10217289	114241	451704	169104	402811	204391	7174838	109925
100	3	7	3	3	400	2.5	31782	126541	47481	10372218	113571	451857	174872	412398	205962	7374444	113744
100	1	3	3	3	400	5	33601	126290	46351	10234611	117000	453520	181797	407272	205537	7246158	96802
100	1	7	5	5	400	5	37644	133571	47055	11036980	124373	483108	200238	432744	223709	7817995	109799
100	2	3	5	5	400	5	34314	135349	51584	10872980	122918	476658	185860	438728	218504	7653055	103641
100	2	7	3	3	400	5	32554	128050	48847	10798849	118656	468824	178634	426141	214307	7670198	103277
100	3	3	3	3	400	5	33945	135998	46958	10994084	123715	483022	174983	433816	217712	7803074	106734
100	3	7	5	5	400	5	27719	109524	44436	8976742	99649	397281	153972	346867	182732	6110902	95840
100	2	5	4	4	500	0	31434	122582	49076	10343442	113952	453474	169442	409618	208056	7323928	97920
100	3	5	4	4	500	0	31673	125506	47044	10490040	116422	462821	191522	416518	208470	7433410	118805

Table 1. Continuation

TP	TM	CO	VT	VA	M	H	C18:1 11t	C18:1 9c	C18:1 11c	C18:2 n6	C20:0	C18:3 n3	CLA 9c, 11t	C20:4 n6
60	1	5	4	4	100	0	862080	12665160	337520	1170520	0	748400	402080	301160
60	3	5	4	4	100	0	1051010	15918257	393267	1289267	0	842099	495129	322535
60	1	3	3	3	200	2.5	577224	8142940	235015	892881	20597	552642	255119	238433
60	1	7	5	5	200	2.5	907402	13458848	346495	1239020	33235	784681	415907	325147
60	2	3	5	5	200	2.5	923103	13828793	351084	1200640	36182	808916	429458	310887
60	3	3	3	3	200	2.5	939307	14029537	353356	1198507	35620	767502	418961	303498
60	3	7	5	5	200	2.5	1166975	17653200	433500	1380350	38900	921325	531375	350825
60	1	3	5	5	200	5	670659	9505951	275683	984707	0	621317	292073	269732
60	1	7	3	3	200	5	722766	10863073	299356	1146059	37288	702278	341473	306776
60	2	7	5	5	200	5	1040224	15771119	385846	1287438	40100	841045	480796	332040
60	3	3	5	5	200	5	1027034	15601201	384412	1301985	37181	855539	474118	320196
60	3	7	3	3	200	5	1055254	16250912	386941	1316166	38312	858746	481493	328302
60	1	5	4	4	300	7.5	660294	9707064	265525	1022301	26836	628548	295492	276361
60	2	5	4	4	300	7.5	928383	14102469	348304	1243960	38033	779776	420752	313716
60	3	5	4	4	300	7.5	1089145	16325632	405750	1365763	38145	854895	484474	326882
60	1	5	4	4	300	0	789223	11451987	298698	1124133	31841	699880	357116	293635
60	1	5	4	4	300	0	728613	10846853	286507	1082120	29747	665520	328880	285000
60	1	5	4	4	300	0	734855	10852763	294763	1082842	25566	673197	328461	286118
60	1	5	4	4	300	0	732321	10779639	284642	1097779	33231	676789	329057	288187
60	1	1	4	4	300	0	470508	6482535	197901	766007	19723	464673	199050	219657
60	1	9	4	4	300	0	512242	7757463	239154	1100537	22886	631221	237020	322523
60	1	5	2	2	300	0	454322	6583201	191960	821436	19490	483839	192987	231631
60	1	5	6	6	300	0	816931	11960297	319604	1128475	32178	713406	372594	301267
60	2	5	4	4	300	0	996174	15195839	362040	1302255	39718	812322	451235	321852
60	2	5	4	4	300	0	1017611	15627168	372528	1318653	40488	837360	471432	325782
60	2	5	4	4	300	0	1016970	15021666	371711	1265167	44944	819711	459069	333311

60	2	1	4	4	300	0	741203	10995673	285582	1091974	30353	673477	336497	287608
60	2	9	4	4	300	0	731226	11193364	300210	1204577	30505	731502	333167	326216
60	2	5	6	6	300	0	1054382	16116651	388283	1339046	45632	859382	487658	332546
60	3	5	4	4	300	0	1115685	16990111	395318	1355764	42164	870610	506216	324184
60	3	5	4	4	300	0	1069387	16506413	387733	1348587	37813	859200	490360	332573
60	3	5	4	4	300	0	1074483	16448886	387302	1373221	44577	856255	503034	328913
60	3	5	4	4	300	0	1097664	16814805	403638	1355638	33235	875383	497852	339973
60	3	1	4	4	300	0	873563	13265523	329470	1176821	32887	747444	396689	299921
60	3	9	4	4	300	0	935503	14450872	362336	1361933	36054	839396	425275	347114
60	3	5	2	2	300	0	905255	13672940	326872	1259752	33208	787383	408819	316188
60	3	5	6	6	300	0	1209533	18062980	437428	1432283	40618	927770	541125	356862
60	1	3	5	5	400	2.5	544597	7728992	224584	872645	20730	531272	233980	241763
60	1	7	3	3	400	2.5	538151	8138844	229738	1021960	25418	593109	240542	283656
60	2	3	3	3	400	2.5	536111	7645867	216926	896748	25400	531548	230148	245259
60	3	3	5	5	400	2.5	936733	14054751	352618	1262332	33491	797843	422469	319925
60	3	7	3	3	400	2.5	1019489	15547429	364624	1351331	38263	843549	457820	332075
60	1	3	3	3	400	5	316174	4276833	134856	582886	14273	354174	125652	174530
60	1	7	5	5	400	5	752714	11339271	311193	1204410	33907	726432	340905	323518
60	2	3	5	5	400	5	750442	11114318	292121	1103750	29495	679470	334785	287109
60	2	7	3	3	400	5	767910	11705551	298257	1218195	32948	727788	348389	316631
60	3	3	3	3	400	5	627970	9318104	250030	1000630	25830	610185	276081	267830
60	3	7	5	5	400	5	1140264	17386307	416683	1425327	52538	900503	524548	341884
60	1	5	4	4	500	0	630998	9306333	253643	1046269	28016	620353	276096	280337
60	2	5	4	4	500	0	904940	13666980	332763	1297655	36225	789896	412651	321213
60	3	5	4	4	500	0	1144040	17327703	415463	1585411	44080	975110	506677	393002
70	1	5	4	4	100	0	1008699	15103456	386485	1267806	0	829204	464466	320777
70	1	3	5	5	200	2.5	927550	13930300	362050	1242250	36300	797425	425525	317875
70	1	7	5	5	200	5	1055051	16040152	334899	1337828	41591	863712	476061	340303
70	2	5	2	2	300	0	1019974	15670337	318594	1354125	38818	848680	457221	328224

70	1	5	4	4	300	7.5	873168	13060980	282523	1216859	34148	758362	389651	307087
70	1	7	3	3	200	2.5	998789	15226915	372000	1302769	37387	826131	452955	324121
70	2	3	3	3	200	2.5	1016324	15550574	373544	1310750	38647	835500	459441	324971
70	2	7	5	5	200	2.5	1170600	17828325	370975	1389025	43150	914525	507875	347525
70	3	7	3	3	200	2.5	1118134	17229881	402299	1352746	42910	867164	477284	323552
70	1	3	3	3	200	5	738211	10952563	251668	1080950	29307	678106	328266	285829
70	1	5	4	4	300	0	971144	14727799	367813	1282288	38475	810395	437030	318341
70	2	3	5	5	200	5	1101990	16728358	345572	1352637	39900	890970	498930	338806
70	2	7	3	3	200	5	1123258	17446939	341682	1379455	42894	883197	496955	331970
70	3	3	3	3	200	5	1060054	16217644	385396	1311149	40604	841634	477609	317450
70	3	5	2	2	300	0	1072980	16471510	330423	1340805	38604	850074	475215	324362
70	3	3	5	5	200	2.5	1076061	16389192	395909	1306768	40480	855227	479545	328131
70	3	7	5	5	200	5	1166275	17738700	425300	1365300	47425	902475	492900	337025
70	1	5	4	4	300	0	967280	14830920	365760	1294013	36880	816187	438307	320533
70	2	5	4	4	100	0	1067373	15814627	401059	1283020	0	844706	473529	317451
70	3	5	4	4	100	0	1108769	16027885	399885	1279308	0	852615	468769	315577
70	1	5	2	2	300	0	676317	10238178	268620	1097888	27776	656396	299333	285578
70	1	5	6	6	300	0	1068691	16077730	329191	1307842	40224	855039	477513	330474
70	1	5	4	4	300	0	986230	14929823	316748	1296210	38544	820669	442767	324341
70	1	5	4	4	300	0	1003639	15347251	321793	1344268	38662	839050	451826	330154
70	1	7	5	5	400	2.5	1080625	16691513	400700	1412500	41163	885363	487038	345738
70	1	3	5	5	400	5	753325	11152655	299876	1106228	29194	686241	332382	291824
70	1	1	4	4	300	0	104399	1454033	51017	231641	0	141608	40399	70844
70	1	9	4	4	300	0	935427	14413507	310453	1366613	36907	845867	418667	353640
70	2	5	4	4	300	7.5	1080170	16625666	394675	1355685	42072	864459	485325	327410
70	3	5	4	4	300	7.5	1116660	17419221	402561	1392594	42482	886389	497492	339446
70	2	5	4	4	300	0	1087163	16811402	392292	1351442	40744	861236	487096	326020
70	2	5	4	4	300	0	1107240	17388173	409053	1396600	43307	894227	500427	340173
70	2	5	4	4	300	0	1099841	17056119	402212	1372649	42967	874675	494291	329801

70	2	5	4	4	300	0	1123086	17419762	349974	1398715	43205	894874	504596	336411
70	2	1	4	4	300	0	145716	1986746	72317	341162	0	202574	58825	104000
70	2	9	4	4	300	0	1088609	17052464	342066	1404371	43232	895523	477364	347404
70	3	5	4	4	300	0	1094490	17175483	396079	1362278	42371	873298	484464	327523
70	3	5	4	4	300	0	1134203	17639593	406085	1394833	42964	896682	498636	335554
70	1	3	3	3	400	2.5	502032	7246849	210839	855252	21104	515228	211441	238010
70	1	5	4	4	500	0	836946	12653371	312786	1207503	32232	738453	373531	304786
70	2	3	5	5	400	2.5	1063693	16141545	384485	1354761	41030	852161	476080	328869
70	2	7	3	3	400	2.5	1068045	16468032	334429	1371344	40002	864800	474397	330606
70	3	3	3	3	400	2.5	894672	13792873	329149	1243955	34336	770291	401881	305828
70	3	7	5	5	400	2.5	1183127	18464603	418759	1457841	44789	930136	506563	351030
70	1	7	3	3	400	5	814713	12522277	315404	1256701	33000	759060	367227	322594
70	2	3	3	3	400	5	726037	10895768	283145	1118027	30060	673362	321127	286616
70	2	7	5	5	400	5	1175995	18237836	424540	1458321	45124	927973	519714	352761
70	3	3	5	5	400	5	1075825	16679375	389563	1347838	40188	858263	484125	326688
70	3	7	3	3	400	5	1117784	17367201	380142	1390104	42351	884134	493194	333381
70	3	5	4	4	300	0	1105515	17269355	399309	1365621	41741	875309	488027	326140
70	3	5	4	4	300	0	1160901	17925629	414993	1398583	44636	899656	509483	335682
70	3	9	4	4	300	0	1252827	19435840	455827	1590760	51493	1009280	537320	387307
70	2	5	4	4	500	0	1114576	17016176	393648	1413976	41280	892176	493696	341456
70	3	1	4	4	300	0	224389	2915020	104940	473919	0	281906	85732	147396
70	3	5	4	4	500	0	1117387	17481868	389539	1407335	41242	893852	500681	337820
70	2	5	6	6	300	0	1110964	17136138	407469	1337370	40564	876177	499731	333148
70	3	5	6	6	300	0	1180938	17982728	422833	1384859	45698	900393	507561	334170
80	2	5	4	4	100	0	1125800	16924800	422120	1339200	0	864160	440640	317200
80	2	5	4	4	100	0	1084320	16167800	353160	1295920	0	866920	440840	319640
80	3	5	4	4	100	0	1242835	18199689	467728	1471029	0	975689	456388	418641
80	3	5	4	4	100	0	1150720	17432240	367600	1369520	0	931120	419000	344080
80	1	5	4	4	100	0	1090920	16033320	418360	1307120	0	854520	471760	338320

80	1	5	4	4	100	0	1116510	16533490	428627	1328549	0	866902	472471	342745
80	1	5	4	4	300	7.5	1074539	16750316	390461	1368592	42829	869303	480526	333947
80	2	5	4	4	300	7.5	1149040	17909787	354693	1387920	41107	900920	488720	340987
80	1	3	3	3	200	2.5	998216	15167035	379688	1264960	36045	805643	436884	314005
80	1	5	4	4	300	7.5	1057373	16513215	391327	1361518	44766	861967	475076	327617
80	1	7	5	5	200	2.5	1219752	18095248	444158	1406708	48193	923441	510000	345817
80	1	7	3	3	200	2.5	1153034	17946798	417281	1403542	45828	888310	495901	332232
80	2	3	5	5	200	2.5	1090200	17001175	352525	1325075	31375	866350	468300	334100
80	2	7	3	3	200	2.5	1123055	17251176	414558	1360673	42166	876829	446231	332668
80	2	3	3	3	200	2.5	1076433	16703761	349612	1318597	43731	855343	464746	318940
80	2	7	5	5	200	2.5	1143209	17373209	365149	1336194	45796	890448	444900	335746
80	3	3	3	3	200	2.5	1129868	17512250	418103	1370500	48912	890912	472618	337853
80	3	7	5	5	200	2.5	1208675	18319600	452475	1470975	45550	958050	424075	358625
80	3	3	5	5	200	2.5	1181465	17740404	433687	1448359	55000	928763	481086	565556
80	3	7	3	3	200	2.5	1241672	18932209	446970	1502836	48090	970761	449836	363582
80	1	3	5	5	200	2.5	1662365	25872956	616872	2155640	65296	1381232	740222	530443
80	1	7	3	3	200	5	1160436	17285317	424411	1438500	44465	914777	501757	357178
80	1	3	3	3	200	5	992970	15284099	368261	1296901	43685	822044	443897	318916
80	1	3	5	5	200	5	1122153	16444703	426955	1376683	42450	892475	493787	343564
80	2	3	3	3	200	5	1089627	17126851	392597	1350881	36373	868015	483851	328776
80	2	7	5	5	200	5	1123266	17165578	435377	1354749	41784	889121	441608	390276
80	2	3	5	5	200	5	1152228	17554356	364035	1366906	44530	894901	491213	344010
80	2	7	3	3	200	5	1109985	17158373	347716	1362507	44254	874642	448687	336239
80	3	3	5	5	200	5	1132637	17602463	431393	1398532	38383	903060	465622	342736
80	3	7	3	3	200	5	1160111	18240121	420724	1433503	47065	918859	430236	343628
80	3	3	3	3	200	5	1194015	18217020	438060	1474695	46530	936675	509580	357690
80	3	7	5	5	200	5	1814199	28616408	656845	2216990	75291	1428932	692718	528301
80	2	5	4	4	300	7.5	1211680	18181213	356480	1408853	41280	917600	498667	329573
80	3	5	4	4	300	7.5	1075490	16976430	386430	1291866	41409	837906	432430	313866

80	3	5	4	4	300	7.5	1070658	17058365	381754	1313223	27601	832532	425927	316027
80	1	7	5	5	200	5	1164332	17608713	363960	1360272	42178	889307	485941	331782
80	1	5	4	4	300	0	1493070	23152040	533645	1934087	57579	1224040	661846	474381
80	1	5	4	4	300	0	1099553	17018816	405092	1352066	45118	871632	486224	326303
80	1	5	4	4	300	0	1117875	17261307	397518	1396818	45122	884568	494746	341479
80	1	1	4	4	300	0	196797	2574684	93648	408027	0	242884	77103	131468
80	1	9	4	4	300	0	1349806	20855880	477191	1592080	55090	1015197	453003	386970
80	1	5	4	4	300	0	1115707	17260587	397893	1382667	45627	886080	489360	339253
80	1	5	4	4	300	0	1098224	17296434	401066	1391395	41566	883566	490171	336829
80	1	5	4	4	300	0	1177329	18376868	434395	1503684	47132	952987	524763	367987
80	1	5	2	2	300	0	908373	14179307	328900	1272693	35627	781327	404547	311187
80	1	5	6	6	300	0	1144560	17726500	429220	1397860	39920	898260	503120	350520
80	1	5	4	4	300	0	640773	9568507	277667	1087147	25787	654240	280520	302987
80	1	5	4	4	300	0	634373	9534587	272400	1085947	21627	645453	280987	296080
80	1	1	4	4	300	0	186631	2421834	83508	380306	0	230671	76598	126724
80	1	9	4	4	300	0	1193935	18673307	424275	1448288	45137	911046	429529	347673
80	2	5	4	4	300	0	1151801	17753669	350066	1378013	47669	897483	474026	334437
80	2	5	4	4	300	0	1210475	18167144	427064	1444294	50475	934261	499371	354395
80	2	5	4	4	300	0	1126493	17621293	420480	1388667	41373	885760	475880	332067
80	1	5	2	2	300	0	948350	14154924	300634	1257155	31993	775815	413835	310106
80	1	5	6	6	300	0	1104393	17132439	418800	1382361	40505	889397	488243	342452
80	2	5	4	4	300	0	1154684	18062158	362461	1411816	44474	917513	480329	377316
80	2	1	4	4	300	0	304040	4338565	143708	625223	0	366166	129834	179083
80	2	9	4	4	300	0	1181729	18340898	415670	1384686	43604	894086	440607	336607
80	2	5	4	4	300	0	1075373	17042471	388614	1317320	41516	851791	444915	319660
80	2	5	4	4	300	0	1074278	17053629	383960	1335099	43722	852781	454026	312212
80	2	5	4	4	300	0	1108613	17502853	397307	1357160	38867	868280	459827	330240
80	2	5	2	2	300	0	1035532	16347873	346809	1316100	39960	831304	447458	314803
80	2	5	6	6	300	0	1108415	17326074	416127	1323271	32308	859043	455017	325224

80	2	5	4	4	300	0	1129705	17918779	402309	1373087	46403	879651	471007	323087
80	2	1	4	4	300	0	308507	4247080	141280	607560	0	361960	126760	181907
80	2	9	4	4	300	0	1157128	18250518	413180	1392026	61311	897639	460026	327856
80	3	5	4	4	300	0	1071128	17098255	389705	1319906	42188	843154	422215	319436
80	3	5	4	4	300	0	1098566	17440303	394908	1317684	48355	853329	438039	314829
80	3	5	4	4	300	0	1076728	17027974	384026	1307775	37364	846821	418543	310358
80	2	5	2	2	300	0	1048047	16557899	324653	1323300	40943	835111	462444	311542
80	2	5	6	6	300	0	1314282	20470993	473215	1518725	44698	1003369	524477	379168
80	3	5	4	4	300	0	1117080	17443267	414280	1313693	43547	849560	431773	319493
80	3	1	4	4	300	0	403064	5810528	180067	757351	17579	444763	172441	218662
80	3	9	4	4	300	0	1116805	17667745	406805	1385517	38322	869597	382886	320752
80	3	5	4	4	300	0	1080918	17155410	390125	1326177	20144	849469	434938	317482
80	3	5	4	4	300	0	1090728	17032708	391148	1314085	45836	854557	433482	319830
80	3	5	4	4	300	0	1076462	16974442	382614	1309901	47182	846733	419960	311934
80	3	5	2	2	300	0	1045793	16448943	320957	1290582	36120	823405	441579	305023
80	3	5	6	6	300	0	1102953	16979275	336242	1300409	48181	840705	417765	314376
80	3	5	4	4	300	0	1084305	17124450	389232	1298795	40662	845258	428821	309152
80	3	1	4	4	300	0	427463	6040805	184349	765597	0	455154	179651	222846
80	3	9	4	4	300	0	1091118	17439079	383026	1347079	42789	858579	379066	335658
80	1	5	4	4	300	0	1072537	16527409	404094	1350067	41597	858685	473664	332054
80	2	3	3	3	400	2.5	963308	15102915	344513	1270575	36645	792608	440168	304665
80	3	5	2	2	300	0	1050622	16564575	311833	1294569	42308	829926	442689	306080
80	3	3	5	5	400	2.5	1123865	17943554	403990	1367045	43890	877182	477556	330848
80	3	5	6	6	300	0	1102828	17032669	401444	1304046	45219	842940	418093	314921
80	1	3	5	5	400	2.5	957090	15146915	351269	1263209	36244	789017	430012	308545
80	1	3	5	5	400	2.5	961990	15093831	358433	1247525	35299	781704	423072	308259
80	1	3	5	5	400	2.5	962015	15039192	365000	1250958	30087	786294	434975	310124
80	1	7	3	3	400	2.5	1080254	16728898	375621	1367708	40227	860027	455686	327367
80	1	3	3	3	400	2.5	692642	10790261	271724	1081642	22090	649291	307724	271851

80	1	7	5	5	400	2.5	1150088	18304625	408038	1423550	51138	902600	493075	333113
80	2	7	5	5	400	2.5	1123242	17817968	399626	1375287	42556	877606	448217	331347
80	2	3	5	5	400	2.5	1042649	16630609	376182	1307662	41679	834701	457836	319241
80	2	7	3	3	400	2.5	1174856	18382604	357460	1450574	43619	918780	484842	343968
80	2	3	5	5	400	5	1085273	17326377	389888	1358065	41935	861712	480025	327519
80	3	7	5	5	400	2.5	1153881	17939030	413520	1347363	43371	864925	412699	329080
80	3	7	3	3	400	2.5	1128188	17687668	374276	1360085	40756	871786	442734	322191
80	3	3	3	3	400	2.5	1028852	16118467	350763	1302281	39830	827993	446437	308037
80	3	7	5	5	400	5	1107662	17729428	394701	1355721	45274	874714	415187	333756
80	1	3	3	3	400	5	747604	11205821	282910	1096164	29284	671679	317769	280336
80	1	7	5	5	400	5	1127556	17851012	406333	1398222	35753	882210	484840	333444
80	1	3	5	5	400	5	975650	15151500	355475	1267963	32825	804263	431950	312700
80	1	7	3	3	400	5	1026289	15987356	356630	1341274	38659	834970	447904	325081
80	2	7	3	3	400	5	1147728	17923399	389252	1412776	43190	892743	482963	335065
80	2	3	3	3	400	5	959174	14821712	340124	1272342	43824	795581	426223	310891
80	2	7	5	5	400	5	1158400	18530782	413759	1431340	49888	909628	465782	334467
80	3	3	3	3	400	5	1032617	16304173	354932	1312489	39872	828346	455842	309008
80	3	3	5	5	400	5	1111300	17551473	397710	1366312	30965	868478	471473	324257
80	3	7	3	3	400	5	1163533	17938116	344977	1366462	43545	874568	456136	319229
80	1	5	4	4	500	0	1053752	16392736	367888	1349184	41048	841568	459800	318520
80	1	5	4	4	500	0	1035541	16207350	363517	1321632	27422	835913	453592	319240
80	2	5	4	4	500	0	1168192	18116431	399250	1423122	41373	898651	491361	337309
80	2	5	4	4	500	0	1143152	18112871	389640	1419081	42804	901616	490495	333703
80	3	5	4	4	500	0	1092473	17335006	374012	1345916	40497	858445	456345	320401
80	3	5	4	4	500	0	1116867	17500024	372169	1364000	44209	866892	454145	320900
100	2	5	4	4	100	0	1038297	15412911	410812	1230772	0	799723	174970	282812
100	2	5	4	4	300	7.5	1107003	16984704	345010	1316704	23322	829329	280359	317834
100	3	5	4	4	300	7.5	1039442	16336186	318379	1274857	42219	814724	201449	298007
100	1	5	4	4	100	0	1066280	15762000	439080	1243280	0	825120	285080	336240

100	1	5	4	4	300	7.5	1627342	25729395	567763	2003342	66526	1265684	520526	479447
100	1	1	4	4	300	0	455329	6296966	212443	814832	39195	474832	184215	225624
100	1	9	4	4	300	0	1178480	18135000	424213	1401813	47147	933080	266653	349400
100	2	5	4	4	300	0	1087398	16982127	401057	1339625	32428	838756	253043	308134
100	2	5	4	4	300	0	1072993	16699020	399101	1292282	33154	830322	241356	310805
100	2	5	4	4	300	0	1082938	17080393	423502	1319895	21639	850151	255974	329154
100	1	3	3	3	200	2.5	1036961	16402420	376493	1287410	47854	827034	364946	306878
100	1	5	4	4	300	0	1065760	16984173	388733	1324933	42773	849600	313307	323720
100	2	3	5	5	200	2.5	1117635	17481133	348793	1326010	42118	890468	297512	344507
100	2	7	3	3	200	2.5	1042305	16274940	330645	1278870	26115	808995	180075	307590
100	3	3	3	3	200	2.5	1046000	16239239	383612	1247478	35597	809299	245015	304672
100	3	7	5	5	200	2.5	1015249	15664229	320075	1210323	46020	803781	108159	299328
100	1	5	4	4	300	0	1067787	16746698	325143	1298551	28106	825701	307415	314591
100	1	7	3	3	200	5	1109627	17365478	409985	1347090	33552	862701	261149	320015
100	2	3	3	3	200	5	1038179	16376284	319910	1280045	26881	814045	313134	300045
100	2	7	5	5	200	5	1065303	16380303	403485	1311566	0	821641	193283	309621
100	3	3	5	5	200	5	1005347	15539802	302822	1171287	43020	769035	204827	283738
100	3	7	3	3	200	5	1047754	16095528	387769	1273492	32367	807829	130281	307477
100	2	5	4	4	300	0	1107556	17424781	404687	1367663	51434	865535	240862	335582
100	2	1	4	4	300	0	618066	8820961	221618	981474	20342	578132	252526	258500
100	2	9	4	4	300	0	1118752	17447718	353128	1371409	34993	867154	163450	343812
100	3	5	4	4	300	0	1067974	16769592	387658	1295579	47934	818579	185829	308632
100	3	5	4	4	300	0	1104693	17049120	408347	1300080	32947	829693	198827	304987
100	3	5	4	4	300	0	1044066	16478250	318421	1279211	26961	811105	188921	301684
100	3	5	4	4	300	0	1156571	17925940	346777	1375628	49834	872532	204545	316904
100	3	1	4	4	300	0	661360	9915300	277030	1037993	0	624053	267512	274284
100	3	9	4	4	300	0	1082301	16762549	327569	1283595	33830	814523	124444	315386
100	1	5	2	2	300	0	1056214	16536314	319438	1279826	44555	816381	372107	293732
100	1	5	6	6	300	0	1072863	16534980	335667	1319137	49196	832059	298569	315392

100	1	5	4	4	300	0	1112803	17437445	402234	1326689	42435	863853	322569	321632
100	1	5	4	4	300	0	1114381	17258033	406421	1331893	36027	851679	336495	324936
100	2	5	2	2	300	0	1041674	16361518	323205	1280840	11752	813433	315583	297107
100	2	5	6	6	300	0	1051010	16275525	324950	1253960	55366	810376	199584	288079
100	1	7	5	5	200	2.5	1127550	17161300	415900	1316600	0	878625	249075	317250
100	1	3	5	5	200	5	1108775	17086525	421175	1312800	0	860525	353325	333850
100	3	5	2	2	300	0	1311572	20290562	400127	1588328	50134	1006087	354749	370428
100	3	5	6	6	300	0	1066391	16519252	319947	1272636	32980	820391	210497	308960
100	1	3	5	5	400	2.5	1128582	17498669	390510	1347687	35510	863781	410211	334938
100	1	7	3	3	400	2.5	1185030	18192252	359089	1390956	59726	889756	341637	329074
100	2	3	3	3	400	2.5	1077594	16901150	326789	1318549	45218	831722	377564	311301
100	2	7	5	5	400	2.5	1146566	17783308	409424	1400564	41203	875514	203396	333271
100	3	3	5	5	400	2.5	1063371	16756978	386940	1299764	28570	821766	272687	313619
100	3	7	3	3	400	2.5	1088308	17020835	329970	1317436	54594	836489	198075	306699
100	1	3	3	3	400	5	1081455	16811777	372668	1326995	49062	841804	431064	323176
100	1	7	5	5	400	5	1151040	18164198	413734	1405100	41629	901654	290990	332707
100	2	3	5	5	400	5	1124289	17808130	336035	1339389	29314	882307	343940	328080
100	2	7	3	3	400	5	1128899	17783027	347592	1381678	45215	873386	268173	325478
100	3	3	3	3	400	5	1163263	18008844	354774	1380983	10169	886868	390037	321320
100	3	7	5	5	400	5	917419	14360038	339085	1114850	23634	720702	126679	266617
100	2	5	4	4	500	0	1073865	17040526	323195	1318733	41020	843378	299036	312303
100	3	5	4	4	500	0	1102311	17208829	334940	1318629	44327	844343	256327	309530

Table 1. continuation

TP	TM	CO	VT	VA	M	H	C20:5 n3	C22:5	C22:6 n3
60	1	5	4	4	100	0	297360	283440	65440
60	3	5	4	4	100	0	316634	305426	81386
60	1	3	3	3	200	2.5	249134	229493	55179

60	1	7	5	5	200	2.5	332059	295980	77500
60	2	3	5	5	200	2.5	303571	287660	77167
60	3	3	3	3	200	2.5	309278	283229	76610
60	3	7	5	5	200	2.5	351175	342625	96725
60	1	3	5	5	200	5	275927	302171	55244
60	1	7	3	3	200	5	314034	282746	73288
60	2	7	5	5	200	5	322587	312463	79851
60	3	3	5	5	200	5	339412	312500	87255
60	3	7	3	3	200	5	329151	307859	76171
60	1	5	4	4	300	7.5	286702	254181	64107
60	2	5	4	4	300	7.5	316950	292884	75168
60	3	5	4	4	300	7.5	329013	308171	84368
60	1	5	4	4	300	0	302140	276465	69196
60	1	5	4	4	300	0	306120	261707	67160
60	1	5	4	4	300	0	301697	271711	67184
60	1	5	4	4	300	0	302181	266140	67211
60	1	1	4	4	300	0	234838	206337	50601
60	1	9	4	4	300	0	334376	291101	77557
60	1	5	2	2	300	0	248564	216886	52832
60	1	5	6	6	300	0	298218	283426	71089
60	2	5	4	4	300	0	325181	296000	83262
60	2	5	4	4	300	0	331723	315815	95142
60	2	5	4	4	300	0	324997	296262	78269
60	2	1	4	4	300	0	301595	267869	78915
60	2	9	4	4	300	0	334046	298177	79882
60	2	5	6	6	300	0	334717	340046	83862
60	3	5	4	4	300	0	329967	312302	87436
60	3	5	4	4	300	0	329413	304747	82267
60	3	5	4	4	300	0	331597	311235	76000

60	3	5	4	4	300	0	340859	317584	87732
60	3	1	4	4	300	0	312185	281179	74159
60	3	9	4	4	300	0	356067	325248	86134
60	3	5	2	2	300	0	324342	295779	84403
60	3	5	6	6	300	0	356349	337678	98329
60	1	3	5	5	400	2.5	262796	226650	57947
60	1	7	3	3	400	2.5	299443	257673	69386
60	2	3	3	3	400	2.5	259696	232385	65030
60	3	3	5	5	400	2.5	327120	302057	81658
60	3	7	3	3	400	2.5	337707	307526	81195
60	1	3	3	3	400	5	192364	165280	39341
60	1	7	5	5	400	5	333995	295063	77500
60	2	3	5	5	400	5	295076	270593	72462
60	2	7	3	3	400	5	323978	287192	84651
60	3	3	3	3	400	5	282296	245681	63081
60	3	7	5	5	400	5	344611	323744	82173
60	1	5	4	4	500	0	290525	260064	68473
60	2	5	4	4	500	0	334538	296924	77936
60	3	5	4	4	500	0	398004	360216	97924
70	1	5	4	4	100	0	313243	303262	77748
70	1	3	5	5	200	2.5	316050	295750	76900
70	1	7	5	5	200	5	333914	334798	105556
70	2	5	2	2	300	0	335663	300706	78990
70	1	5	4	4	300	7.5	318054	284255	73436
70	1	7	3	3	200	2.5	327844	298734	81784
70	2	3	3	3	200	2.5	333088	300235	78368
70	2	7	5	5	200	2.5	338675	324625	87775
70	3	7	3	3	200	2.5	323985	301507	81776
70	1	3	3	3	200	5	293201	261558	66452

70	1	5	4	4	300	0	325043	295719	78957
70	2	3	5	5	200	5	333259	319602	82289
70	2	7	3	3	200	5	332333	326621	101212
70	3	3	3	3	200	5	322262	293525	81757
70	3	5	2	2	300	0	325262	295497	79685
70	3	3	5	5	200	2.5	322424	302778	82424
70	3	7	5	5	200	5	331675	319125	88950
70	1	5	4	4	300	0	327733	296440	80107
70	2	5	4	4	100	0	313412	300667	79686
70	3	5	4	4	100	0	309423	300462	82731
70	1	5	2	2	300	0	297300	258858	67551
70	1	5	6	6	300	0	329230	306257	81671
70	1	5	4	4	300	0	329508	299292	79108
70	1	5	4	4	300	0	337003	304548	79786
70	1	7	5	5	400	2.5	350388	320900	84088
70	1	3	5	5	400	5	303462	267333	69764
70	1	1	4	4	300	0	87123	74857	17329
70	1	9	4	4	300	0	358640	323187	86947
70	2	5	4	4	300	7.5	333561	307148	80826
70	3	5	4	4	300	7.5	337135	311485	85215
70	2	5	4	4	300	0	328133	304279	81342
70	2	5	4	4	300	0	339333	316440	84387
70	2	5	4	4	300	0	331642	307219	82834
70	2	5	4	4	300	0	338596	315086	85364
70	2	1	4	4	300	0	121267	107393	27366
70	2	9	4	4	300	0	348503	323338	85563
70	3	5	4	4	300	0	328954	305960	82517
70	3	5	4	4	300	0	336997	314125	82741
70	1	3	3	3	400	2.5	256017	218213	56376

70	1	5	4	4	500	0	313675	278084	74140
70	2	3	5	5	400	2.5	335239	305892	81985
70	2	7	3	3	400	2.5	336404	305656	80566
70	3	3	3	3	400	2.5	314157	280119	75687
70	3	7	5	5	400	2.5	348102	325794	89218
70	1	7	3	3	400	5	330389	290940	77110
70	2	3	3	3	400	5	297599	261142	68424
70	2	7	5	5	400	5	350560	327164	86343
70	3	3	5	5	400	5	330075	302138	82163
70	3	7	3	3	400	5	329746	305828	81918
70	3	5	4	4	300	0	330565	307362	82950
70	3	5	4	4	300	0	336252	314265	84000
70	3	9	4	4	300	0	386387	360600	96507
70	2	5	4	4	500	0	346792	314720	83664
70	3	1	4	4	300	0	162617	143409	34591
70	3	5	4	4	500	0	341355	311519	83311
70	2	5	6	6	300	0	333954	311508	86518
70	3	5	6	6	300	0	334407	315757	82761
80	2	5	4	4	100	0	328160	310120	80800
80	2	5	4	4	100	0	330880	294800	65880
80	3	5	4	4	100	0	365126	348311	94796
80	3	5	4	4	100	0	338640	323560	91160
80	1	5	4	4	100	0	321880	306840	71640
80	1	5	4	4	100	0	325294	308588	94196
80	1	5	4	4	300	7.5	336500	309053	81671
80	2	5	4	4	300	7.5	337120	314107	85520
80	1	3	3	3	200	2.5	318422	284729	76628
80	1	5	4	4	300	7.5	328779	308132	81439
80	1	7	5	5	200	2.5	333441	310743	81238

80	1	7	3	3	200	2.5	335350	310966	81754
80	2	3	5	5	200	2.5	324950	310050	89200
80	2	7	3	3	200	2.5	329216	297980	84829
80	2	3	3	3	200	2.5	325224	302045	86761
80	2	7	5	5	200	2.5	327438	308433	88358
80	3	3	3	3	200	2.5	334309	311912	85382
80	3	7	5	5	200	2.5	351750	328500	95250
80	3	3	5	5	200	2.5	759672	337424	86843
80	3	7	3	3	200	2.5	348418	378224	128672
80	1	3	5	5	200	2.5	527118	487931	131305
80	1	7	3	3	200	5	343782	324178	88708
80	1	3	3	3	200	5	316123	293202	78606
80	1	3	5	5	200	5	341906	315322	88267
80	2	3	3	3	200	5	327910	301672	80373
80	2	7	5	5	200	5	333744	309397	81809
80	2	3	5	5	200	5	343193	316460	86089
80	2	7	3	3	200	5	322000	304224	84851
80	3	3	5	5	200	5	338557	333085	83458
80	3	7	3	3	200	5	338819	325930	85462
80	3	3	3	3	200	5	350745	325065	83925
80	3	7	5	5	200	5	534976	491359	136044
80	2	5	4	4	300	7.5	331933	308867	84613
80	3	5	4	4	300	7.5	310013	295409	77570
80	3	5	4	4	300	7.5	314605	295920	79389
80	1	7	5	5	200	5	343168	322723	80421
80	1	5	4	4	300	0	469659	433391	114809
80	1	5	4	4	300	0	327513	307276	84803
80	1	5	4	4	300	0	335881	308422	87248
80	1	1	4	4	300	0	141767	124346	31787

80	1	9	4	4	300	0	377458	366060	99732
80	1	5	4	4	300	0	332187	304387	82680
80	1	5	4	4	300	0	336974	311921	82289
80	1	5	4	4	300	0	385803	354316	97750
80	1	5	2	2	300	0	316807	281973	76547
80	1	5	6	6	300	0	341980	465400	78900
80	1	5	4	4	300	0	303680	275987	70587
80	1	5	4	4	300	0	311493	270413	73227
80	1	1	4	4	300	0	134392	116213	27761
80	1	9	4	4	300	0	341987	316928	87085
80	2	5	4	4	300	0	335894	315629	84715
80	2	5	4	4	300	0	346087	322582	86261
80	2	5	4	4	300	0	328173	308307	83560
80	1	5	2	2	300	0	312449	280376	76726
80	1	5	6	6	300	0	343043	311213	79889
80	2	5	4	4	300	0	345829	318316	85487
80	2	1	4	4	300	0	200027	173555	45701
80	2	9	4	4	300	0	326904	308898	81479
80	2	5	4	4	300	0	317608	290745	81176
80	2	5	4	4	300	0	320702	298901	81947
80	2	5	4	4	300	0	318533	303000	79920
80	2	5	2	2	300	0	316013	287237	76977
80	2	5	6	6	300	0	318301	306161	79866
80	2	5	4	4	300	0	318040	304403	79007
80	2	1	4	4	300	0	196093	168347	45027
80	2	9	4	4	300	0	330413	308105	82780
80	3	5	4	4	300	0	315047	292805	76537
80	3	5	4	4	300	0	308105	292671	83987
80	3	5	4	4	300	0	311735	293020	81033

80	2	5	2	2	300	0	312121	285919	77394
80	2	5	6	6	300	0	370852	360081	91248
80	3	5	4	4	300	0	312360	296147	79240
80	3	1	4	4	300	0	229271	201298	50274
80	3	9	4	4	300	0	325812	298926	79409
80	3	5	4	4	300	0	309102	297469	81325
80	3	5	4	4	300	0	306964	295252	79725
80	3	5	4	4	300	0	314640	287960	81175
80	3	5	2	2	300	0	308816	279204	73171
80	3	5	6	6	300	0	306886	299376	76691
80	3	5	4	4	300	0	307510	291841	79126
80	3	1	4	4	300	0	229530	204148	51181
80	3	9	4	4	300	0	316237	295961	80447
80	1	5	4	4	300	0	332752	303678	78362
80	2	3	3	3	400	2.5	307193	279075	78023
80	3	5	2	2	300	0	303003	277284	76301
80	3	3	5	5	400	2.5	317681	303541	80449
80	3	5	6	6	300	0	308563	290344	76887
80	1	3	5	5	400	2.5	307127	283595	80460
80	1	3	5	5	400	2.5	306505	280522	76070
80	1	3	5	5	400	2.5	307152	301567	71580
80	1	7	3	3	400	2.5	327950	295534	81180
80	1	3	3	3	400	2.5	285000	248836	66209
80	1	7	5	5	400	2.5	348000	315413	89125
80	2	7	5	5	400	2.5	319601	298716	83728
80	2	3	5	5	400	2.5	317301	293818	77998
80	2	7	3	3	400	2.5	345550	316968	81913
80	2	3	5	5	400	5	332965	301663	81973
80	3	7	5	5	400	2.5	314527	301729	80510

80	3	7	3	3	400	2.5	323932	296729	81874
80	3	3	3	3	400	2.5	317356	284207	78615
80	3	7	5	5	400	5	328706	301095	85933
80	1	3	3	3	400	5	284455	257836	70888
80	1	7	5	5	400	5	333432	311296	82321
80	1	3	5	5	400	5	314775	281138	80313
80	1	7	3	3	400	5	320852	290681	76807
80	2	7	3	3	400	5	338244	306067	81516
80	2	3	3	3	400	5	316251	280690	75491
80	2	7	5	5	400	5	336600	308263	80422
80	3	3	3	3	400	5	316083	283586	75632
80	3	3	5	5	400	5	319282	296597	84530
80	3	7	3	3	400	5	315083	293095	78445
80	1	5	4	4	500	0	323888	296592	81496
80	1	5	4	4	500	0	318329	285046	77228
80	2	5	4	4	500	0	331457	305573	82643
80	2	5	4	4	500	0	335430	304879	81933
80	3	5	4	4	500	0	322373	291808	78068
80	3	5	4	4	500	0	317687	292394	78892
100	2	5	4	4	100	0	305030	285030	84000
100	2	5	4	4	300	7.5	352332	313342	85116
100	3	5	4	4	300	7.5	305967	283309	75508
100	1	5	4	4	100	0	330200	318720	90840
100	1	5	4	4	300	7.5	463000	425921	129513
100	1	1	4	4	300	0	245570	209141	50711
100	1	9	4	4	300	0	326920	308853	92027
100	2	5	4	4	300	0	312763	287064	83599
100	2	5	4	4	300	0	307758	284430	80658
100	2	5	4	4	300	0	307043	282348	88393

100	1	3	3	3	200	2.5	303966	284473	76229
100	1	5	4	4	300	0	316547	304560	82467
100	2	3	5	5	200	2.5	316305	319286	100616
100	2	7	3	3	200	2.5	329085	301275	92310
100	3	3	3	3	200	2.5	297448	281507	76149
100	3	7	5	5	200	2.5	293433	275846	82587
100	1	5	4	4	300	0	301515	289940	74924
100	1	7	3	3	200	5	313567	286284	81134
100	2	3	3	3	200	5	302284	288776	70537
100	2	7	5	5	200	5	318561	298081	83838
100	3	3	5	5	200	5	287921	267871	72946
100	3	7	3	3	200	5	299518	278382	76538
100	2	5	4	4	300	0	326801	306168	87044
100	2	1	4	4	300	0	268408	251895	73658
100	2	9	4	4	300	0	321893	282376	98537
100	3	5	4	4	300	0	302158	281526	79776
100	3	5	4	4	300	0	307147	288000	79307
100	3	5	4	4	300	0	297211	278461	73289
100	3	5	4	4	300	0	323615	293635	81807
100	3	1	4	4	300	0	279366	254759	70667
100	3	9	4	4	300	0	296444	274928	85190
100	1	5	2	2	300	0	306702	275472	74742
100	1	5	6	6	300	0	311451	288745	78000
100	1	5	4	4	300	0	314997	290475	72201
100	1	5	4	4	300	0	319612	291197	74368
100	2	5	2	2	300	0	297140	272782	74156
100	2	5	6	6	300	0	305208	274396	73723
100	1	7	5	5	200	2.5	316275	306175	85000
100	1	3	5	5	200	5	326200	301650	100050

100	3	5	2	2	300	0	369231	334729	91251
100	3	5	6	6	300	0	307132	286430	78159
100	1	3	5	5	400	2.5	317475	292425	74241
100	1	7	3	3	400	2.5	323178	299770	82407
100	2	3	3	3	400	2.5	308368	291737	76850
100	2	7	5	5	400	2.5	324236	302481	82945
100	3	3	5	5	400	2.5	308794	290435	79403
100	3	7	3	3	400	2.5	301459	286128	77519
100	1	3	3	3	400	5	316790	286693	82656
100	1	7	5	5	400	5	331115	296617	81629
100	2	3	5	5	400	5	314214	311845	86621
100	2	7	3	3	400	5	314428	296755	83027
100	3	3	3	3	400	5	321543	299792	78670
100	3	7	5	5	400	5	263095	239812	67732
100	2	5	4	4	500	0	309275	286462	78789
100	3	5	4	4	500	0	310127	286837	78311
